# Srsf1 and Elavl1 act antagonistically on neuronal fate choice in the developing neocortex by controlling TrkC receptor isoform expression

**DOI:** 10.1093/nar/gkad703

**Published:** 2023-09-11

**Authors:** A Ioana Weber, Srinivas Parthasarathy, Ekaterina Borisova, Ekaterina Epifanova, Marco Preußner, Alexandra Rusanova, Mateusz C Ambrozkiewicz, Paraskevi Bessa, Andrew G Newman, Lisa Müller, Heiner Schaal, Florian Heyd, Victor Tarabykin

**Affiliations:** Charité Universitätsmedizin Berlin, Institute of Cell Biology and Neurobiology, Charitéplatz 1, 10117 Berlin, Germany; Freie Universität Berlin, Institute of Chemistry and Biochemistry, Takustr. 6, 14195, Berlin, Germany; Charité Universitätsmedizin Berlin, Institute of Cell Biology and Neurobiology, Charitéplatz 1, 10117 Berlin, Germany; Charité Universitätsmedizin Berlin, Institute of Cell Biology and Neurobiology, Charitéplatz 1, 10117 Berlin, Germany; Research Institute of Medical Genetics, Tomsk National Research Medical Center of the Russian Academy of Sciences, 634009, Tomsk, Russia; Charité Universitätsmedizin Berlin, Institute of Cell Biology and Neurobiology, Charitéplatz 1, 10117 Berlin, Germany; Freie Universität Berlin, Institute of Chemistry and Biochemistry, Takustr. 6, 14195, Berlin, Germany; Charité Universitätsmedizin Berlin, Institute of Cell Biology and Neurobiology, Charitéplatz 1, 10117 Berlin, Germany; Research Institute of Medical Genetics, Tomsk National Research Medical Center of the Russian Academy of Sciences, 634009, Tomsk, Russia; Charité Universitätsmedizin Berlin, Institute of Cell Biology and Neurobiology, Charitéplatz 1, 10117 Berlin, Germany; Charité Universitätsmedizin Berlin, Institute of Cell Biology and Neurobiology, Charitéplatz 1, 10117 Berlin, Germany; Charité Universitätsmedizin Berlin, Institute of Cell Biology and Neurobiology, Charitéplatz 1, 10117 Berlin, Germany; Heinrich Heine Universität Düsseldorf, Institute of Virology, Medical Faculty, Universitätsstr. 1, 40225 Düsseldorf, Germany; Heinrich Heine Universität Düsseldorf, Institute of Virology, Medical Faculty, Universitätsstr. 1, 40225 Düsseldorf, Germany; Freie Universität Berlin, Institute of Chemistry and Biochemistry, Takustr. 6, 14195, Berlin, Germany; Charité Universitätsmedizin Berlin, Institute of Cell Biology and Neurobiology, Charitéplatz 1, 10117 Berlin, Germany; Institute of Neuroscience, Lobachevsky State University of Nizhny Novgorod, 603950, Nizhny Novgorod Oblast, Russia

## Abstract

The seat of higher-order cognitive abilities in mammals, the neocortex, is a complex structure, organized in several layers. The different subtypes of principal neurons are distributed in precise ratios and at specific positions in these layers and are generated by the same neural progenitor cells (NPCs), steered by a spatially and temporally specified combination of molecular cues that are incompletely understood. Recently, we discovered that an alternatively spliced isoform of the TrkC receptor lacking the kinase domain, TrkC-T1, is a determinant of the corticofugal projection neuron (CFuPN) fate. Here, we show that the finely tuned balance between TrkC-T1 and the better known, kinase domain-containing isoform, TrkC-TK+, is cell type-specific in the developing cortex and established through the antagonistic actions of two RNA-binding proteins, Srsf1 and Elavl1. Moreover, our data show that Srsf1 promotes the CFuPN fate and Elavl1 promotes the callosal projection neuron (CPN) fate *in vivo* via regulating the distinct ratios of TrkC-T1 to TrkC-TK+. Taken together, we connect spatio-temporal expression of Srsf1 and Elavl1 in the developing neocortex with the regulation of TrkC alternative splicing and transcript stability and neuronal fate choice, thus adding to the mechanistic and functional understanding of alternative splicing *in vivo*.

## Graphical Abstract

**Graphical Abstract:**
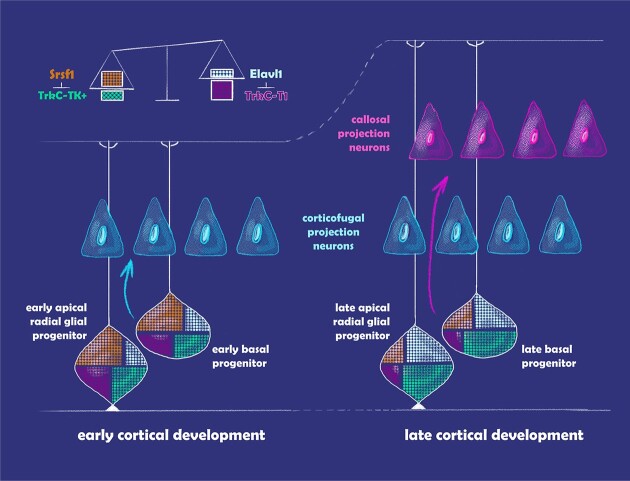

## INTRODUCTION

The plethora of projection neuron subtypes in the cerebral neocortex is generated during embryonic development by a transient pool of neural progenitor cells (NPCs) (reviewed in (1–3)). While the ultimately six-layered neocortex is one of the defining features of mammals, the numbers and ratios between subtypes are species-specific, and an increased complexity of this organization is generally accepted to have enabled heightened cognitive function in primates (4–6). Abnormalities in the positioning, morphology or numbers of cortical neuron subtypes often result in developmental neuropsychiatric disorders that impair cognitive, sensory and motor functions (7–9). Therefore, unravelling how NPCs generate the correct numbers of the different projection neuron subtypes is pivotal for understanding the development of both the healthy and the diseased neocortex.

The differentiative behavior, and hence fate choices, of cortical NPCs is governed by their cellular identity, which, in turn, is governed by a precise transcriptomic composition. We currently still only have a partial understanding of the factors determining the specific transcriptome in NPCs and its causal relationships to what neuron subtypes these cells produce. In general, RNA-binding proteins (RBPs) are known to considerably impact the transcriptome (10,11) as they regulate all steps of RNA processing. This eventually determines the abundance and composition of mature transcripts, and some RBPs are known to exhibit strikingly diverse and dynamic spatio-temporal expression profiles in the developing cortex (12–14).

Among the many processes that RBPs control, alternative splicing and differential stabilization of (pre-m)RNAs are highly prevalent in the brain, and the protein diversity that these processes enables is thought to have contributed to the evolutionary expansion of cortical complexity (15–23). Furthermore, RBPs regulating alternative splicing (termed splicing factors in the following, in short, SFs) have been implicated in several steps of cortical projection neurogenesis, such as NPC maintenance, neurogenic division, and neuron migration and morphology acquisition (reviewed in (24), and see (25)). However, to date, we have very limited knowledge on how SFs contribute to neuronal subtype fate acquisition, despite some SFs being expressed in patterns that are specific to NPCs or during key stages in the production of distinct neuron subtypes (12–14).

We previously showed that the NPC levels of an alternatively spliced isoform of the neurotrophin-3 receptor TrkC determine the acquisition of corticofugal projection neuron fate (CFuPN) at the expense of callosal projection neuron fate (CPN) (26). This control is crucial due to the striking functional differences of the two neuron subtypes in the cortical circuitry, with CFuPNs projecting outside of the cerebral cortex and CPNs within. Based on this finding, we hypothesized that CFuPN-CPN fate determination through TrkC-T1 is the result of dynamic RBP-controlled mRNA isoform expression in the developing cortex. Here, we show that the CFuPN-CPN fate choice, as dictated by the levels of TrkC isoforms, is orchestrated by two key SFs, Srsf1 and Elavl1. We first report that TrkC-T1 and TrkC-TK+ levels are finely regulated in NPC- and neuron-specific ratios in the developing cortex. Through *in vitro* and *in vivo* dosage manipulations, we uncover that the RBPs Srsf1 and Elavl1 act as antagonistic regulators of TrkC mRNAs, ensuring the NPC- and neuron-specific balance between the two receptor transcript and protein variants, and thereby the generation of the correct proportion of CFuPNs to CPNs. Additionally, we show that the combination of Srsf1 and Elavl1 levels defines distinct, cell type-specific environments for regulating the levels of TrkC isoforms. Finally, we identify an exonic splicing enhancer in the T1-specific exon 13A as an Srsf1-dependent *cis-*regulatory element. This is the first demonstration of Srsf1 and Elavl1 co-regulating binary neuronal fate decisions in the developing mammalian cortex and provides an *in vivo* example to underline the importance of RBPs in neurogenesis.

## MATERIALS AND METHODS

### Immortalized cell line culture and treatment

N2A and HEK293T cells were, unless otherwise specified, cultured in DMEM (4.5 g glucose/l, supplied with GlutaMAX L-glutamine, Gibco, 10566) supplemented with 10% fetal bovine serum (Biochrom) and penicillin-streptomycin (1000 U/ml, Gibco, 15140122). For transfection, we used the TurboFect reagent (Thermo Scientific, R0533), according to the instructions of the producer. In the respective experiments, actinomycin D was applied for the indicated length of time (final concentration: 20 μg/ml, Sigma-Aldrich, A9415).

### Primary neuron preparation and nucleofection

Primary cortical neurons were cultured in dishes that were coated overnight at room temperature with poly-L-lysine (final concentration 10 μg/ml, Sigma-Aldrich, A-005-M) and laminin (final concentration 0.2 μg/ml, Sigma-Aldrich, L2020). For harvesting the neurons, pregnant dams were sacrificed at the indicated days of embryonic development, the embryos released from the uterine horns, and their cortical hemispheres excised while removing the hippocampal anlage and detaching from the ganglionic eminences. After rinsing in HBSS – (Gibco, 14175095), the cortex pieces were dissociated using a 0.3125% trypsin solution in HBSS– (Gibco, 15090046) for 15 min at 37°C and then treated with DNase I (final concentration of 0.05 mg/ml, Roche, 10104159001) for 2 min at room temperature. The cortical cells were resuspended in embryonic neuron culture medium (1 ml 50× B27 supplement without vitamin A, Gibco, 12587010, 500 μl GlutaMAX supplement, 500 μl of penicillin-streptomycin stock, added to 48 ml Neurobasal medium, Gibco, 12348017).

Nucleofection of primary neurons with expression constructs was performed using the Mouse Neuron Nucleofector Kit according to the producer-supplied protocol (Lonza, VPG-1001) together with the Amaxa Nucleofector device (Nucleofector 2b, Lonza, AAB-1001). We nucleofected 1 μg of plasmid for every 10^6^ cortical cells and then plated them in a medium consisting of 1 ml SM1 supplement (provided with BrainPhys medium) and 500 μl GlutaMAX supplement (Gibco, 35050061), added to 48.5 ml BrainPhys medium, (STEMCell Technologies, 05792).

### Cell and tissue staining procedures

For chromogenic RNA *in situ* hybridization, embryonic brain slices were incubated with digoxigenin (DIG)-labeled probes. The primers used to generate the RNA probes contained SP6 RNA polymerase promoters and had the sequences listed in the table below.

**Table:** 

ISH probe primer	Sequence
Srsf1 fw	GGCTACGACTACGACGGCTACCGG
Srsf1 rv	ATTATTTAGGTGACACTATAGGATTGTACTGAGTAAAGGAAAACTGT
Elavl1 fw	GTTAGACAGATGGGGAGTGTGTT
Elavl1 rv	ATTATTTAGGTGACACTATAGTGCTCACAAGAAGGGATGCG

For the transcription, 1 μg linearized plasmid were mixed with 2 μl 10× transcription buffer, 2 μl of 100 mM DTT, 0.5 μl RNase inhibitor, 2 μl of 10× DIG labeling mix (Roche, 11277073910), 20 U SP6 RNA polymerase (Thermo Scientific, EP0131), to 20 μl with RNase-free MilliQ water. All containers and solutions used prior to and during the RNA probe hybridization were treated against RNase contamination by heating them at 200°C for two hours. For the prehybridization, 1 ml of hybridization solution (50% deionised formamide p.a, 0.1 mg/ml yeast tRNA, 10% dextran sulphate, 1:50 dilution of Denhardt's solution, Thermo Fisher, 750018 and a 1:10 dilution of a salt solution containing 2 M NaCl, 50 mM EDTA, 100 mM Tris–HCl pH 7.5, 50 mM NaH2PO4·2H2O, 50 mM Na2HPO4) was applied per slide and the slides incubated at 65°C for 1 h. 200 μl of the probe mixture (50 μl probe solution denatured in 100 μl formamide for 5 min at 95°C) were applied per slide and slides were incubated as above overnight. Unbound probe was removed by three washes in a stringent washing solution (50% formamide, 1× SSC, 0.1%, Tween-20), after which the slides were washed with MABT buffer (100 mM maleic acid, 150 mM NaCl, 0.1% Tween-20, pH 7.5), then blocked for 1 h at room temperature in a 2% blocking reagent solution (Roche, 11 096 176 001) with 10% sheep serum in 1x MABT. A 1:1500 solution of alkaline phosphatase-coupled anti-DIG antibodies (Roche, 11093274910) in MABT was applied overnight at 4°C. Unbound antibodies were washed off at room temperature in 1x MABT buffer, then in pre-staining solution (4 ml of 5M NaCl, 10 ml of 1 M MgCl_2_, 20 ml of 1 M Tris pH 9.5, 0.2 ml of Tween 20 in 166 ml of MilliQ water). Slides were then incubated at 37°C in staining solution with chromogenic AP substrate until the colored precipitate could be observed. Staining solution: 0.8 ml of 5 M NaCl, 2 ml of 1 M MgCl_2_, 4 ml of 1 M Tris pH 9.5, 13.2 ml H_2_O, 40 μl Tween-20, 40 μl of NBT (1000× = 100 mg/ml in 70% DMSO), 40 μl of BCIP (1000× = 50 mg/ml in 100% DMSO), supplemented up to 40 ml with 10% PVA in H_2_O. Coverslips were mounted with Entellan (Sigma-Aldrich, 107960).

For immunofluorescent staining, 50 μm brain sections were blocked for 30 min in blocking solution (10% horse serum, 0.1% Triton X-100 in PBS), then incubated overnight with primary antibody in blocking solution with gentle shaking. On the second day, sections were washed 4 × 10 min in an excess of PBS, then fluorophore-coupled secondary antibodies were applied for 4 h at room temperature. Sections were then mounted onto SuperFrost Plus glass slides with ImmuMount (Thermo Scientific, 9990402).

### Image acquisition, processing and quantification

The slides resulting from *in situ* hybridization were imaged on a Zeiss BX60 system. Linear modifications of brightness were performed using ImageJ software. For the immunofluorescently stained tissue preparations, we used a Leica Sp8 confocal laser scanning system with a DMI6000CSB microscope (BioSupraMol facility at Freie Universität Berlin). When analyzing fate acquisition, we marked around 100–300 GFP-positive cells per analyzed electroporation site for Ctip2 and Satb2 or Cux1 co-expression and counted each dual labeling using the Cell Counter plugin in ImageJ. Counting was performed blinded. For each electroporated litter, brain sections were matched for anteroposterior and lateromedial position of the electroporation site. To quantify the fold change in fate, individual brains were compared to the mean percentage of double positive cells of that fate in the littermate controls.

### Fluorescence-activated cell sorting (FACS)

Primary cortical cells, prepared as described above, were resuspended in PBS, stained with the APC-coupled anti-prominin-1 or isotype control antibody plus propidium iodide in PBS on ice for 30 min, and then sorted for PI and APC signal using a BD FACSCanto or FACSMelody sorter. PI-negative cells were collected in two separate tubes, depending on the presence or absence of APC signal. The collection medium was based on our Neurobasal culture medium and supplemented with recombinant murine EGF (final concentration: 40 ng/ml, ImmunoTools, 12343406) and FGF2 (final concentration: 40 ng/ml, ImmunoTools, 12343623). Cells were then pelleted by centrifugation and the pellets snap-frozen in liquid nitrogen for downstream applications.

### Quantitative real-time PCR

For tissues, RNA was extracted using the ReliaPrep RNA extraction kit (Promega, Z6212) and reverse transcribed into first strand cDNA with an oligo(dT) primer (Promega, C1101) and the Promega GoScript reverse transcription system (A5000). TaqMan RT-qPCR was performed using the FastAdvanced Master Mix (Thermo Fisher, 4444557) on a StepOne Plus RT-qPCR cycler (Thermo Fisher/Applied Biosystems, 4376600). Reactions were set up according to the master mix protocol using the equivalent of 25 ng reverse transcribed RNA per 10 μl reaction. Reactions were performed in technical quadruplicates and the number of biological replicates indicated in the figures. The following TaqMan probes were used: for TrkC-T1, VIC-tagged Mm01317842_m1 and for TrkC-TK+, a custom-designed exon junction spanning FAM-tagged probe (AR47VWU), both from Thermo Fisher.

For cultured cells, the RNA was extracted using a standard phenol-chloroform extraction procedure employing the TRIzol reagent (Ambion/Invitrogen, 15596018) and followed by a DNase treatment (Lucigen, D9905K) and a phenol-chloroform-isoamyl alcohol extraction (Carl Roth, X985.1). The resulting RNA was reverse-transcribed using MMuLV reverse transcriptase (Enzymatics/Qiagen, P7040L) using either oligo(dT) primers (RT-qPCR) or gene-specific reverse primers (splicing-sensitive RT-PCR), according to the producer's protocol. For SYBR Green RT-qPCR, we used the Promega GoTaq RT-qPCR system (A6001), according to the producer's protocol. Amplification efficiency was calculated using the Thermo Fisher qPCR efficiency calculator.

**Table:** 

RT-qPCR primer	Sequence
Srsf1 fw	CCCTTCGCCTTCGTTGAGTTCG
Srsf1 rv	GAAACTCTACCCGCAGCCGG
Elavl1 fw	TCGGGATAAAGTAGCAGGACACAG
Elavl1 rv	CTGGAGTCTCAAGCCGTTCAGT
Hprt fw	CAACGGGGGACATAAAAGTTATTGGTGGA
Hprt rv	TGCAACCTTAACCATTTTGGGGCTGT
Oaz1 fw	AAGGACAGTTTTGCAGCTCTCC
Oaz1 rv	TCTGTCCTCACGGTTCTTGGG

### siRNAs and 2′-MOEs

siRNAs were purchased from Dharmacon/Horizon Discovery (ON-TargetPlus siRNA pools), transfected into N2a cells using the RotiFect reagent (Roth, P001.3) at a final concentration of 25 nM. The siRNA pool against Srsf1 was L-040886-01-0005, the one against Elavl1 was L-053812-00-0005, and the one against Elavl2 L-065473-01-0005. 2′-MOEs were purchased from Microsynth. The sequence for the 2′-MOE against E 13A-3 was CAGGTTCCTCATATATATAG. The control for the 2′-MOE experiment was the non-targeting siAllstar, sequence: (UUC UCC GAA CGU GUC ACG U)TT.

### Radioactive splicing-sensitive PCR

Splicing-sensitive PCRs were performed with transcript variant-discriminating primers either with radioactive labelling of primers or without. Radioactive RT-PCRs were performed as described (27). Briefly, 200 ng of the primer binding to both transcript variants were labelled with 32P-Γ-ATP (Hartmann Analytic, SRP-501) using 10 units T4 PNK (Molox) for 1h at 37°C, and then purified and precipitated using the PCI protocol as described above. Primer pellets were resuspended in 80 μl H_2_O, and 1 μl of this labelled primer was used per 20 μl PCR reaction. After the PCR, products were mixed 1:1 with formamide loading buffer, denatured alongside the marker (NEB, N3032S) for 5 min at 95°C, and 5 μl were applied to a denaturing polyacrylamide-urea gel (7 M urea, 8% polyacrylamide in 0.5× TBE). Once the desired degree of resolution was reached, gels were fixed, transferred to Whatman paper, vacuum-dried and finally assembled with a photostimulatable phosphor plate in photographic cassettes. The plates were then imaged on a GE Healthcare Typhoon 7000 FLA Phosphorimager and the result quantified using the ImageQuant TL software, version 8.1.

### UV crosslinking of radioactive RNA probes to nuclear extract proteins

T25 flaks with N2a cells at 80% from confluency were transfected with the empty vector (pCAGIG) or Srsf1 overexpression vector (pCAG-Srsf1). Transfection efficiency was confirmed by epifluorescence microscopy. Of these cells, nuclear extracts were prepared by nuclear-cytosolic fractionation in RNase-free buffers. While on ice, cells were washed twice with ice-cold PBS, then gently resuspended in a volume equal to five times the packed cell volume of the low salt CTX buffer (10 mM HEPES, 1.5 mM MgCl_2_, 10 mM KCl). After five min, the same volume of CTX buffer with 0.2% NP-40 was added and the suspension was gently pipetted up and down, then left on ice for another five min. Nuclei were pelleted by centrifugation at 6500 rpm for three min (4°C). The supernatant (cytosolic fraction) was collected in a separate tube. After washing once with PBS, nuclei were resuspended in one packed nuclei volume of the high salt NX buffer (20 mM HEPES, 400 mM KCl, 1,5 mM MgCl_2_, 0.2 mM EDTA, 25% glycerin). A 5 M NaCl solution was added at 1:12 of the total volume in order to reach a concentration of 400 mM NaCl. Cells were then incubated on ice for five minutes, vortexing vigorously throughout. The mixture was then subjected to three cycles of freezing at -80°C and thawing on ice. Finally, the resulting suspension was centrifuged at 14 000 rpm for 20 min and the resulting supernatant (nuclear extract) was transferred to a fresh tube. Nuclear and cytosolic extracts were subjected to Western blotting with antibodies against cytoplasmatic proteins (vinculin) and nuclear proteins (hnRNP L) to confirm successful fractionation.

### Western blotting

To collect tissue or cells for protein extraction, we used RIPA buffer (50 mM Tris pH 8, 150 mM NaCl, 0.1% SDS, 1% NP-4, 0.5% sodium deoxycholate), freshly supplied with protease inhibitors (1× protease inhibitor cocktail (Sigma, P8340), 5 μg/ml pepstatin, 2.5 mM sodium orthovanadate, 10 mM benzaminidine, 10 μg/ml leupeptin, 1 mM β-glycerophosphate and 5 mM NaF). From the cleared lysates, around 40 μg total protein were denatured for 5 min at 95°C in 1× protein sample buffer (for 5×, 312.5 mM Tris, pH 6.8, 50% glycerol, 20% SDS, 20% β-mercaptoethanol, 2% Bromophenol Blue) and were then applied per lane of a 8–10% Tris-glycine gel. Proteins were separated and transferred to PVDF membranes (Immobilon-P, Millipore, IPVH000010) in transfer buffer (3:5 water, 1:5 methanol, 1:5 transfer buffer stock, consisting of 144.2 g glycine, 30.3 g Tris base, 1 g SDS supplemented to 2 l with double-distilled water) using the Mini-Protean Tetra system (BioRad, 1658003EDU). Membranes were blocked for 1h at RT with 5% BSA in TBS-T (10 mM Tris, pH 8, 150 mM NaCl, 0.05% v/v Tween-20) and incubated with primary antibody solutions in TBS-T over night at 4°C. On the following day, membranes were washed with TBS-T and incubated in secondary antibody solution (HRP-coupled, Jackson Immunoresearch) in TBS-T, washed again and developed using the Lightning ECL Plus reagent (Perkin Elmer, NEL104001EA). Signals were quantified using the ImageLab 6.0 software (BioRad).

### Mouse care and experimentation

Experiments involving mice were performed in accordance with animal experimentation licenses granted to the research groups by the Landesamt für Gesundheit und Soziales, Berlin. Mice of the NMRI strain were housed, bred and operated on in the animal facility of Charité Universitätsmedizin Berlin. *In utero* electroporation was performed as described in (28), using a PicoPump PV820 (World Precision Instruments) and a CUY21 electroporator (Bex Co. Ltd.). Following surgery, animals were kept under observation until full recovery and the embryos collected at the pregnancy stages indicated in the experiments.

### Antibodies

**Table:** 

Antibody	Antibody source	Concentration
goat anti-GFP	Rockland, 600-101-215	1:1000 in IF
rat anti-Ctip2	Abcam, 18465	1:300 in IF
rabbit anti-Satb2	custom preparation for Tarabykin research group	1:300 in IF
rabbit anti-Cux1	Santa Cruz (discontinued)	1:100 in IF
mouse anti-Elavl1	Santa Cruz, sc-5261	1:300 in IF
mouse anti-Srsf1	Thermo Fisher, 32-4500	1:1000 in WB
mouse anti-hnRNP L	Santa Cruz, 4D11	1:10000 in WB
rabbit anti-pan-TrkC	Cell Signaling, 3376	1:2000 in WB, 1:100 in IF
mouse anti-GAPDH	HyTest, 5G4cc	1:100 000 in WB
rat anti-Prominin-1, clone 13A4	eBioscience, 17-1331-81	1:200 for FACS
rat IgG1 isotype control	eBioscience, 17-4301-81	1:200 for FACS
Fluorophore-coupled donkey secondary antibodies	Dianova	1:300–1:1000 in IF
HRP-coupled secondary antibodies	Jackson Immunoresearch	1:5000 in WB

### Bioinformatic analysis of RNA sequencing data

For the analysis of single cell datasets, E 14, E 16 and E 18 snRNAseq raw data provided by (29) (GSE153164) was aligned to GRCm38 using cellranger 7.0 with ‘–include-introns’. Count matrices were then imported into Seurat v4 and quality filtered to remove cells containing <5% mitochondrial transcripts and nFeature_RNA < 1000 and nFeature_RNA < 5000. Datasets were integrated using SCT, followed by RunPCA(npcs = 30), RunUMAP(), FindNeighbors(dims 1:20, k.param = 10) and FindClusters(algorithm = 1, resolution 0.3). Markers were then found using FindAllMarkers, and manually annotated according to known biology. Cells belonging to the pyramidal lineage were then subset into a new Seurat object for plotting. For plotting expression of *Srsf1* and *Elavl1* in progenitor cells, cells were subset if they had >0 expression of either *Pax6* or *Tbr2*. Raw *Srsf1* and *Elav1* values were then fit to a negative binomial with ‘celltype’ (*Tbr2-*positive, *Pax6-*positive, *Tbr2/Pax6*-positive) and ‘stage’ (E 14, E 16, E 18) as interaction terms using the MASS package in R. Coefficients from this model can be seen in the table in Supplementary Figure 6D. For stage-specific differences in *Srsf1* and *Elavl1* expression distributions, we used a non-parametric Wilcoxon signed-rank (paired) test to assess whether their population mean ranks differ by cell type. N and *p* values from the Wilcoxon signed rank test can be found summarized per stage in the table in Supplementary Figure 6E and in detail for each cell type and stage in Supplementary Figure 6F. *P* value adjustment for multiple testing was performed with the Benjamin-Hochberg method.

### Statistical analysis

Statistical analysis for laboratory experiments was performed using the Prism software (GraphPad), in accordance with the nature of the experimental setup and employing the tests indicated in the experiments. First, fulfilment of the assumptions required for statistical testing was verified by interrogating whether samples were taken from normally distributed and equal-variance populations with the Shapiro–Wilk and *F* test, respectively. Based on the resulting information and the type of experimental setup, the statistical test was chosen with, if needed, the appropriate corrections. Paired tests were chosen when comparing gene expression between control and experimental animals from the different litters, because cortical differentiation is highly dynamic and small differences in the exact developmental time point can affect overall gene expression. ANOVA post-hoc tests were chosen based on whether samples were compared pairwise or all with a control sample. A detailed description of the statistical test decisional tree employed by GraphPad Prism can be found in the software's documentation (https://www.graphpad.com/guides/prism/latest/statistics/index.htm).

## RESULTS

### Two alternative isoforms of TrkC, TrkC-T1 and TrkC-TK+, are expressed in a stage- and cell type-specific manner in the developing cortex

**Figure 1.**
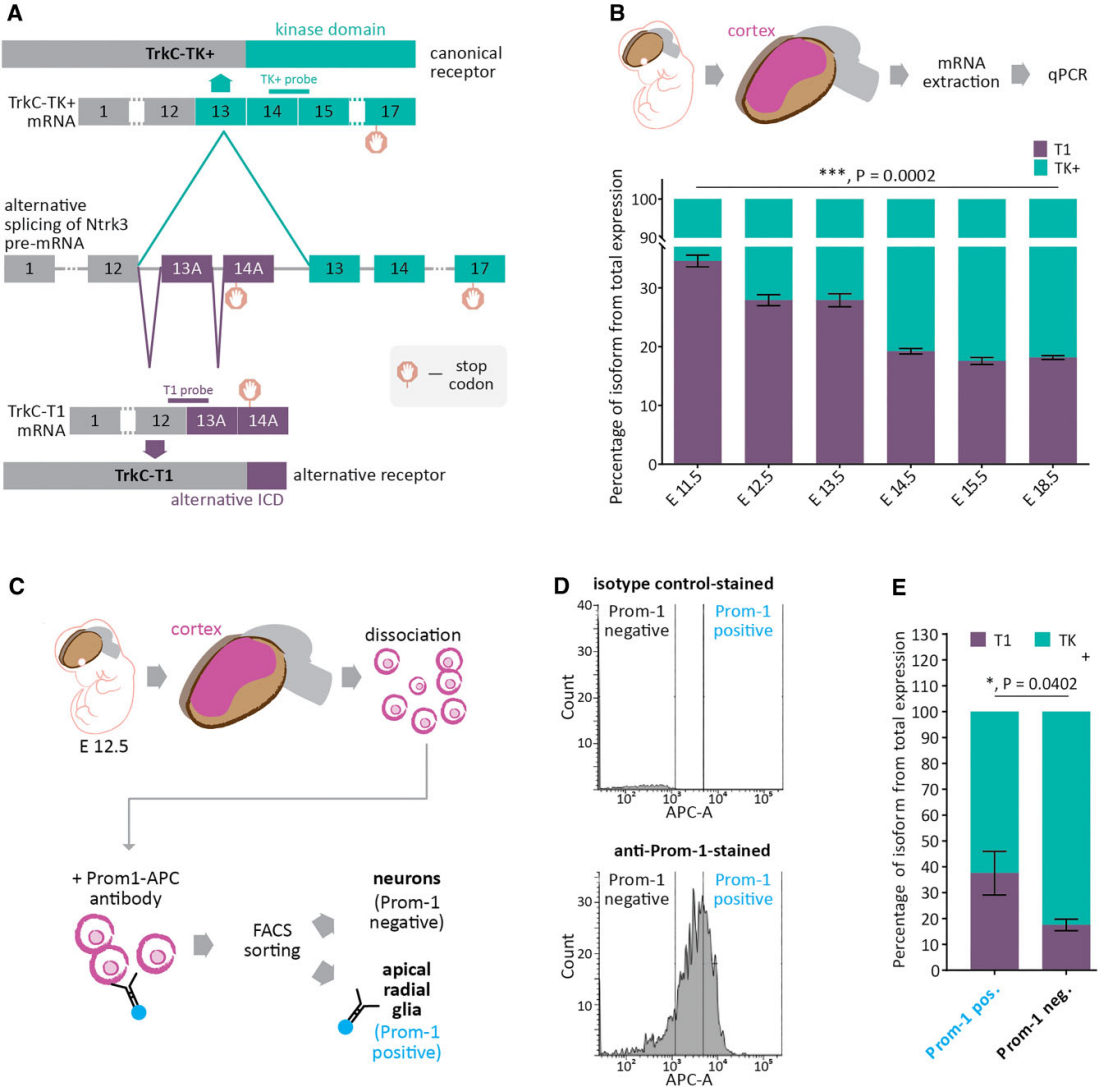
The balance between the two TrkC alternative splicing isoforms TK+ and T1 is regulated in a developmentally dynamic and cell type-specific manner. (**A**) Alternative splicing of the TrkC (Ntrk3) pre-mRNA produces the T1 and TK+ receptor variants. Two groups of mutually exclusive exons (13A-14A and 13–17) give rise to the distinct 3′ termini of the TrkC-TK+ and TrkC-T1 transcript variants. Correspondingly, these translate to distinct intracellular domains at the C-termini of the protein isoforms, giving rise to either a kinase domain (TK+) or a catalytically inactive domain (T1). Stop codons are indicated and demarcate the start of variant-specific 3′ UTRs. Binding sites for the probes used in RT-qPCR are indicated at the respective exon junctions. (**B**) The balance between TrkC-TK+ and TrkC-T1 changes during cortex development. In RNA prepared from cortices of increasing embryonic age, TaqMan quantitative real-time PCR for the two TrkC isoforms shows that the balance between TK+ and T1 shifts in favor of TK+ as cortex development progresses from embryonic day E 11.5 to E 18.5. *N* = 4. Bars: mean percentage of isoform from total TrkC expression (T1 plus TK+) ± SD. P value derived from unpaired, two-tailed Student's *t* test with Welch's correction. (**C–E**). The balance between TrkC-TK+ and TrkC-T1 in the developing cortex is cell-type specific. (**C**) Primary cortical cells from whole E 12.5 embryo litters were sorted into neuronal and stem cell populations by FACS after labelling with an anti-prominin-1 antibody (Prom-1) and collected for further analysis. (**D**) Example APC versus count plots used to distinguish viable Prom-1 positive and negative cells. Gating strategy according to signal from isotype control-stained cells. Complete gating strategy is presented in Supplementary Figure 1B. (**E**) RT-qPCR was performed on mRNA from the sorted neocortical cells, as described in (B). *N* = 3. *P* value derived from paired, two-tailed Student's *t* test. Pairing efficiency between Prom-1 + and Prom-1- results: *r* = 0.9962.

In Parthasarathy *et al.* (26), we characterized two TrkC splice variants that result in the isoforms T1 and TK+ (Figure 1A), and how the finely tuned levels of TrkC-T1 steer the CFuPN-CPN fate choice. Based on these findings, we hypothesized that the quantity of each receptor variant and, thereby, their ratio, is precisely regulated during cortex development. To test this, we assessed the TrkC-T1 to TrkC-TK+ transcript ratio by multiplex TaqMan RT-qPCR in cortical tissue across key stages of corticogenesis (Figure 1B). We found that, as the cortex develops, the ratio of T1 and TK+ mRNA in bulk cortical tissue gradually shifts in favor of the latter variant, with T1 decreasing from a proportion of around 35% in the total TrkC transcript quantity at embryonic day (E) 11.5 to around 15% at E 18.5 (Figure 1B). This is reminiscent of our previous observations on the protein level (26), and suggests that the regulation of the TrkC protein isoform ratio is controlled through differential mRNA isoform expression, caused, for example, by either different stabilities of the transcript isoforms through their different 3′ UTRs or due to alternative splicing.

Based on our observation that the T1 to TK+ ratio gradually changes during cortex development, we wondered whether this effect could be due to ratios specific to the various cortical cell types, whose numbers change in development and thereby may contribute to the shifting ratios observed in the cortical tissue in bulk. At early developmental stages, the cortex consists of a multitude of NPCs and then, as these NPCs divide asymmetrically, becomes gradually enriched in neurons (30). We therefore investigated whether cortical NPCs and neurons exhibit specific ratios of the two TrkC transcript variants. To this end, we sorted primary cortical cells at E 12.5 by FACS using prominin-1 as a marker for apical radial glial cells (aRGCs), a type of cortical NPC. Given that, at E 12.5, the cortex consists primarily of aRGCs and the neurons they produce by direct neurogenic divisions (30,31), this allowed our sorting paradigm to discriminate between NPCs and neurons (Figure 1C–E, Supplementary Figure 1A). We observed a significant difference in the ratio of T1 to TK+ between prominin-1-positive (aRGs) and -negative cells (neurons) (Figure 1E). In neurons, the percentage of T1 of total TrkC transcripts is around 15%, less than half of the percentage seen in NPCs. This indicates a cell type-specific balance between the two transcript variants. Since neurons exhibit a T1 to TK+ ratio that is strongly shifted in favor of TK+, their gradually rising numbers may explain the developmentally increasing dominance of this isoform in the cortex at large. Taken together, we find that the ratio of the TrkC transcript variants is maintained at specific levels in cortical NPCs and neurons.

### Srsf1 and Elavl1 regulate TrkC alternative splicing antagonistically

To address the mechanistic basis for differential TrkC isoform expression, we first considered a potential involvement of micro RNAs (miRNAs) in establishing the levels of TrkC-T1 and TrkC-TK+. However, when we interrogated the 3′ UTR sequences of T1 and TK+ with the miRNA binding site prediction tool TargetScan (32,33) (Supplementary Figure 1B), we could not identify any miRNAs whose expression patterns in the developing cortex are in line with the observed patterns of T1 and TK+ expression (26).

**Figure 2.**
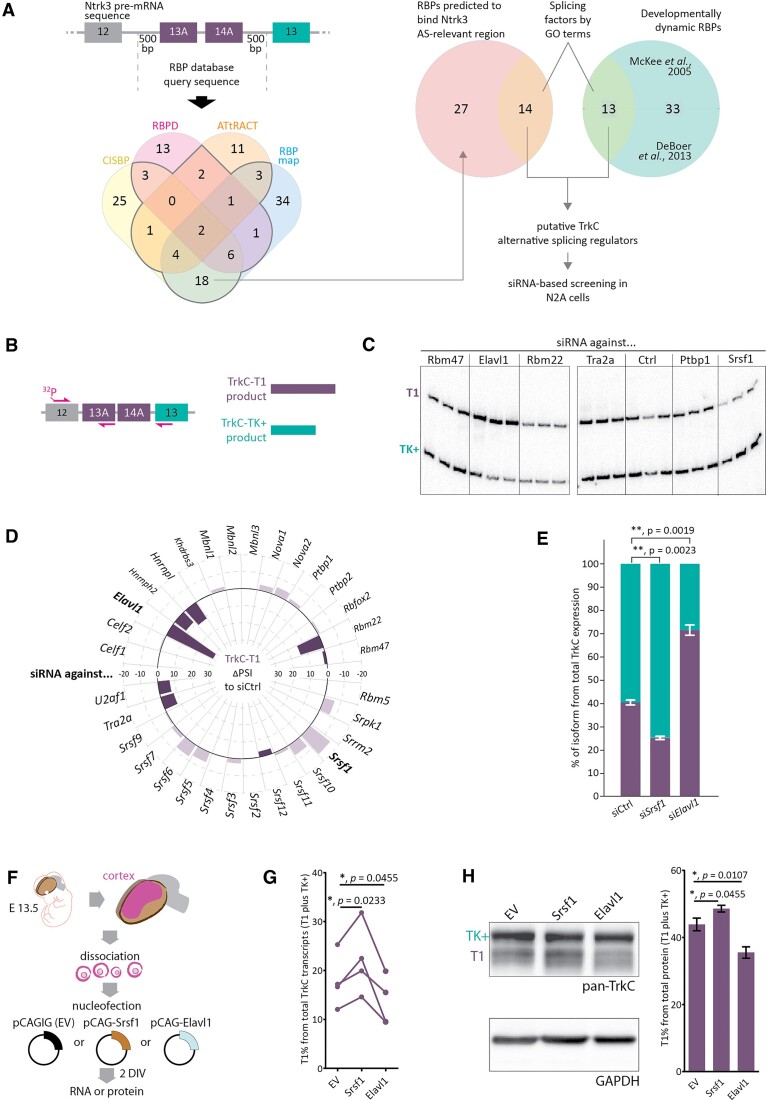
Elavl1 and Srsf1 regulate TrkC alternative splicing in N2A cells and in primary cortical neurons. (**A**) Selection of splicing factors (SFs) and other RNA-binding proteins (RBPs) with potential involvement in TrkC alternative splicing. **B**-**E**. Elavl1 and Srsf1 control TrkC alternative isoform expression. (**B**) Strategy for radioactive splicing-sensitive RT-PCR for evaluating the TrkC-T1 and TrkC-TK+ splicing event. TrkC AS was assessed in N2A cell samples where RBPs defined in (A) were knocked down using siRNAs. (**C**) Exemplary result of a radioactive splicing-sensitive RT-PCR for TrkC-T1 and TrkC-TK+ on RNA from N2A cells treated with the indicated siRNAs. Percentage of TrkC-T1, as represented in (D) and (E), was quantified using a Phosphorimager and the ImageQuant TL software. Ctrl – siCtrl. (**D**) Summary plot for all tested RBPs and their effect on the proportion of the TrkC-T1 transcript variant normalized to TrkC-T1 percentage in the control siRNA samples. Gray dotted circles graduate the plot, indicating increases (positive values, outside the zero circle) or decreases (negative values, inside the zero circle) in TrkC-T1 percentage as compared to the siCtrl samples. Error bars were omitted for clarity. Statistically significant changes (si*Srsf1* and si*Elavl1* samples) are represented separately with the corresponding descriptive and analytical statistical information in (E). (**E**) siRNA-mediated knockdown of Elavl1 or Srsf1 in N2A cells changes the ratio of TrkC-T1 to TrkC-TK+ significantly. *N* = 3. Bars: mean percentage of isoform from total TrkC expression (T1 plus TK+) ± SD. *P* values derived from Brown-Forsythe and Welch ANOVA with Dunnett's T3 multiple comparison *post-hoc* test. Overall *P* value: 0.0002. (**F**) Strategy for modulating SF levels in cortical neurons. Cortices from full litters of E 13.5 embryos were microdissected, dissociated into primary cortical cells and nucleofected with Srsf1 or Elavl1 expression plasmids, or empty expression constructs (pCAGIG = EV). Nucleofected cells were cultured for two days *in vitro* (DIV), after which total RNA or protein were extracted. (**G**) Srsf1 and Elavl1 alter transcript variant ratio of TrkC-T1 and TrkC-TK+ in cortical neurons. RT-qPCR on material from the nucleofected, cultured primary cortical cells, percentage of TrkC-T1 from total TrkC transcripts (T1 plus TK+) is shown. Lines represent paired replicates from the same experiment (cortical cells from one full litter split into the three nucleofection conditions). *N* = 4. *P* values from one-way ANOVA; overall *P* value: 0.0046. (**H**) Western blot of samples from (F), probed with a pan-TrkC antibody, which detects TrkC-TK+ (130 kDa) and TrkC-T1 (100 kDa). GAPDH was detected as a loading control. *N* = 4; *P* values from one-way ANOVA with Dunnett's T3 multiple comparison post-hoc test; overall *P* value: 0.0278.

Based on these findings, we hypothesized that the developmental regulation of the TrkC transcript ratio in the neocortex likely occurs at the level of alternative splicing. Many RNA-binding proteins (RBPs) have been shown before to be crucial for corticogenesis (25,34–36), fulfilling manifold roles and exhibiting variable expression patterns (12). We therefore wondered whether RBPs could contribute to the cortical regulation of TrkC alternative splicing. To identify such RBPs, we searched for those developmentally dynamic SFs which are also predicted to bind in the region of the TrkC pre-mRNA that is relevant to the alternative splicing outcome (Figure 2). We found 32 splicing factors (SFs) that fulfilled these requirements by querying both putative RBP binding sites in the TrkC pre-mRNA, as predicted by four online tools (CISBP (37), RBPDB (38), ATtRACT (39) and RBPmap (40)), and SFs found to be dynamically transcribed in the developing cortex (12,13) (Figure 2A).

To test whether any of these SFs regulate TrkC transcript variant balance (Figure 2B–E), we examined the impact of individual factors by knocking them down with an established siRNA library in N2A cells (41,42), and then assessing the resulting ratio of T1 to TK+ using a radioactive, splice variant-discriminating RT-PCR (Figure 2B and C). Of the 32 tested SFs, Srsf1 and Elavl1, which are strongly expressed in wild type N2A cells (Supplementary Figure 2A), had, by far, the largest impact on TrkC alternative splicing. The Elavl1 knockdown increased the proportion of T1 in the total TrkC transcript pool by over 70% of its levels in the control sample and the knockdown of Srsf1 decreased it by around 50% (Figure 2D, E and Supplementary Figure 2A and B).

The ELAV-like family of RNA binding proteins is comprised of four representatives in mice and humans. Previous research has shown that Elavl2, Elavl3, and Elavl4 share a number of properties, especially in terms of functional redundancy in the nervous system (43). To test whether this particular subgroup of RBPs has an impact on TrkC alternative splicing, we knocked down Elavl2 in N2a cells and assessed the effect on TrkC AS by splicing-sensitive RT-PCR (Supplementary Figure 2C). The knockdown did not significantly change TrkC-T1 levels in the total TrkC-T1 + TrkC-TK+ transcript pool, as opposed to the knockdown of Elavl1, which, as seen in previous experiments, increased TrkC-T1 levels compared to the control siRNA sample.

To investigate a potential cross-regulation of Srsf1 and Elavl1, we altered the protein levels of each of the factors by overexpressing or knocking them down in N2a cells. We then monitored the resulting Srsf1 protein levels by Western blotting (Supplementary Figure 3A) and the Elavl1 protein levels by immunofluorescence (Supplementary Figure 3B). We observed no significant changes of neither Srsf1 nor Elavl1 under these conditions, except for the ones caused, for each of the proteins, by the overexpression plasmid encoding it and the knockdown construct directed against it (Supplementary Figure 3A–C). This indicates that Srsf1 and Elavl1 operate independently from one another to regulate TrkC AS and, potentially, other splicing events.

In order to test if Elavl1 and Srsf1 control TrkC alternative splicing in cortical cells, we isolated primary cortical cells from E 13.5 embryos, overexpressed either Srsf1 or Elavl1 in these cells, cultured them for two days *in vitro* (DIV), and then quantified the TrkC isoform ratios on the transcript and protein level (Figure 2F–H). As before, we saw an increase in the proportion of TrkC-T1 when overexpressing Srsf1. Conversely, the proportion of T1 decreased when overexpressing Elavl1. We could observe these effects both via RT-qPCR (Figure 2G) and Western blotting (Figure 2H). This showed that, indeed, Srsf1 and Elavl1 influence TrkC alternative splicing in primary cortical cells as seen in N2A cells.

### The TrkC-T1-specific exon 13A harbors a splicing enhancer regulated by Srsf1

**Figure 3.**
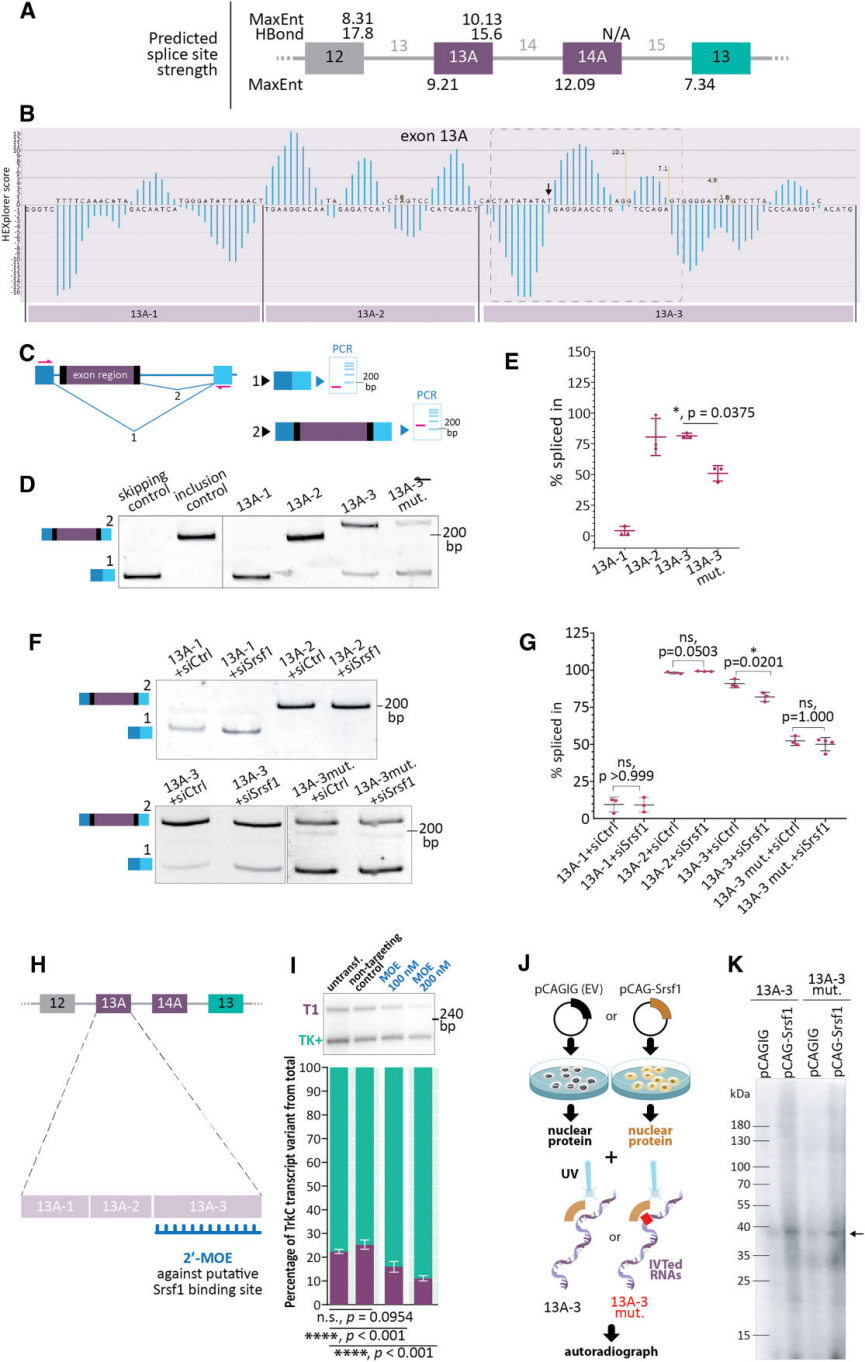
TrkC transcript levels are regulated by an Srsf1-dependent exonic splicing enhancer element in the first TrkC-T1-specific exon, exon 13A. (**A**) Splice site strength prediction of the *Ntrk3* primary transcript. (**B**) Bioinformatic analysis of exon 13A suggests its subdivision in three major splicing-regulatory regions. The arrow points to a nucleotide predicted by HEXplorer to be of particular importance for conferring the splicing enhancer properties to fragment 13A-3. The dotted box marks the region most strongly impacted by this nucleotide and is shown magnified in Supplementary Figure 3B, along with the predicted effects of mutating this nucleotide. **C**-**E**. Minigene analysis of exon 13A splicing regulatory regions. (**C**) Splicing reporter used to assess enhancing or silencing properties of exon 13A fragments. The skipping and inclusion control vectors are described in (47). (**D**) The TrkC-T1 exon 13A reporter vectors were transfected into N2A cells and the splicing outcome assessed by RT-PCR. The mutation in 13A-3 predicted to disrupt Srsf1 binding impedes the splicing enhancing ability of this element, leading to a significant reduction of exon inclusion, as quantified in E. *N* = 3; P values derived from Brown-Forsythe and Welch ANOVA test with Šidak's post-hoc multiple comparisons test. Overall *P* value: <0.0001. The inclusion product of the 13A-3 reporter is larger due to the larger insert size (see B). (**E**) Quantification of (D). (**F**) To assess the involvement of Srsf1 in TrkC alternative splicing, TrkC-T1 exon 13A reporter vectors were transfected into N2A cells together with siRNAs as indicated, and the splicing outcome assessed by RT-PCR. *N* = 3. *P* values derived from ordinary ANOVA test with Šidak's post-hoc multiple comparisons test. Overall *P* value: <0.0001. (**G**) Quantification of (F). (**H, I**) Blocking the putative Srsf1 binding site in exon 13A of the TrkC pre-mRNA leads to a decrease in TrkC-T1 formation. A 2′-MOE antisense oligonucleotide complementary to the putative Srsf1 binding site in exon 13A (H) was transfected into N2a cells and its effect on TrkC alternative splicing assessed by RT-qPCR (I). *N* = 3; *P* values derived from ordinary one-way ANOVA with Šidak's *post-hoc* multiple comparisons test. Overall *P* value: <0.0001. (**J, K**). Radioactively labelled, *in vitro* transcribed RNA probes of the exon 13 A-3 fragment (E 13A-3) or of the same fragment with the mutation described in Supplementary Figure 4B (E 13A-3 mut.) were crosslinked by UV irradiation to nuclear extract proteins from N2a cells transfected either with an Srsf1 overexpression construct (pCAG-Srsf1) or with the empty vector (pCAGIG). Arrow: Srsf1 band (see also Supplementary Figures 3A and 4G).

We next wished to understand which sequence elements contribute to the regulation of TrkC AS. The cassette exons of interest, 13A and 14A (Figure 1A), are flanked by large introns (see Figure 3A, 51.6 kb for intron 13 between exons 12 and 13A, 1.5 kb for intron 14 between exons 13A and 14A, and 40 kb between exons 14A and 13). Therefore, in order to examine alternative splicing regulation, we first predicted the splice site strength of the cassette exons and the flanking constitutive exons (termed 12 and 13). Using the HBond and MAXENT algorithms (44,45), we assessed the splice site score of the 3′ and 5′ splice sites (Figure 3A). The analysis revealed a strong 5′ splice site for exon 12, the last constitutive exon shared by TrkC-T1 and TrkC-TK+, and similar splice site scores for the 3′ splice site of exon 13A (MaxEnt score of 9.21) and 13 (MaxEnt score of 7.34). As no transcript variants have been documented in which only exon 14A is included while exon 13A is skipped (Ensembl genome browser, Howe *et al.*, 2021), the splicing decision between exon 13 and exon 13A likely determines TrkC isoform expression. Given the rather similar splice site strength of the competing 3′ splice sites, this splicing decision is likely controlled through additional *trans*-acting factors that can contribute to cell type-specific splicing patterns. For a first detailed analysis, we have chosen the TrkC-T1-determining exon 13A and used the HEXplorer tool (46) to predict regulatory regions. The resulting probability profile revealed three main putative *cis-*acting regions (Figure 3B). The 5′ region (fragment 1, 13A-1) most likely acts as a splicing silencer, the middle region (fragment 2, 13A-2) as an enhancer, and the last one (fragment 3, 13A-3) contained both potentially enhancing and potentially silencing regions. To determine whether these predictions translate into functional roles and identify potential splicing factor binding sites, we tested the three fragments using a splicing reporter vector (Figure 3C, (47)). Consistent with the predictions, fragment 1 from exon 13A favored exon skipping and fragment 2 exon inclusion (Figure 3D and E). Fragment 3 proved to act as an exonic splicing enhancer as well, albeit less potent.

The analysis of the nucleotides essential for the maintenance of the splicing-regulatory properties of fragment 3 with HEXplorer suggested that a single nucleotide substitution could severely disrupt the ability of this fragment to act as an enhancer (Supplementary Figure 3A). To confirm this potential splicing-regulatory element by a second, independent algorithm, we analyzed the sequence of fragment 13A-3 using the ESEfinder tool (48). We found three GA-rich (GAR) elements predicted to be bound by Srsf1 with high probability (Figure 3B and Supplementary Figure 3B). Additionally, when analyzing the sequence with the mutation predicted to be disruptive by HEXplorer (Supplementary Figure 3A), ESEfinder did not detect any putative Srsf1 binding at this site. Indeed, introducing this mutation in the fragment 3 splicing reporter strongly reduced exon inclusion (Figure 3D and E), underscoring the importance of this region in exon 13A inclusion and hence TrkC-T1 formation. To test whether Srsf1 is indeed required for the inclusion of the exon 13A-3, we expressed the reporter vector harboring this sequence and simultaneously knocked down Srsf1 (Figure 3F). This resulted in a significant reduction of exon inclusion (Figure 3G), confirming that this enhancer is responsive to Srsf1 levels, which, in turn, control TrkC-T1 formation. Importantly, the GAR-mutated reporter vector 13A-3mut did not respond to Srsf1 knock down, suggesting a direct and sequence-specific role of Srsf1 in controlling this splicing event (Figure 3F, G). Additionally, the knockdown did not impact the splicing reporters containing the other two fragments of exon 13A (Figure 3 F and G), suggesting regulation through the 13A-3 sequence.

We also investigated whether the inclusion of exon 13A is influenced by Elavl1 levels. To this end, we used knockdown or overexpression of Elavl1 in cells transfected with the splicing reporters (Supplementary Figure 4C and D). Neither decreasing nor increasing Elavl1 levels changed the splicing behavior of any of the three exon 13A fragments (Supplementary Figure 4D). However, the mutated reporter E 13A-3 mut responded to overexpressing Elavl1, but not to its knockdown. We did not pursue this avenue further, as the mutated sequence is an artificially generated one, and therefore likely not present in a wild type setting.

Given the known role of Elavl1 in regulating mRNA stability, we also tested whether Elavl1 differentially affects the stability of TrkC-T1 and TrkC-TK+. To test this, we knocked down Elavl1 in N2A cells and then inhibited transcription using actinomycin D, monitoring the ratio of TrkC-T1 to TrkC-TK+ after three and six hours of treatment (Supplementary Figure 4F). After the knockdown, we observed an increase of TrkC-T1 in the total transcript quantity similar to the siRNA knockdown experiments (DMSO + siCtrl versus DMSO + siElavl1, compare to Figure 2D and E), but, at the same time, a decrease in the proportion of TrkC-T1 after six hours of actinomycin D treatment as compared to the vehicle control (DMSO + siElavl1). This suggests two modes of action of Elavl1. On the one hand, it controls splicing of TrkC pre-mRNA and suppresses the generation of the TrkC-T1 isoform independent of the exon 13A sequences that we assayed in our reporter vectors. On the other hand, once the TrkC-T1 transcripts are generated, Elavl1 reduces the stability of this isoform, which is in line with the presence of several strong predicted Elavl1 binding sites in the 3′ UTR of TrkC-T1 (Supplementary Figure 3C).

To further understand the mechanism of TrkC AS regulation by Srsf1, we designed a 2′-MOE (antisense oligonucleotide) complementary to region 3 of exon 13A (Figure 3H), which we showed to contain an Srsf1-dependent element (Figure 3F–G). Upon transfecting this 2′-MOE into N2a cells (Figure 3I), the proportion of TrkC-T1 dropped in an ASO-dose dependent manner to around half of its level in the control samples at the highest concentration, showing that this enhancer element is crucial for the control of this alternative splicing event also in the endogenous context. Next, we *in vitro* transcribed radioactively labelled RNA probes with sequences corresponding to E 13A-3 and E 13A-3mut (Figure 3J). We performed UV crosslinking (Figure 3K) using these probes and nuclear extracts obtained from N2a cells that had been transfected with either the Srsf1 overexpression plasmid or the empty vector (Supplementary Figure 4G). Overexpressing Srsf1 clearly increased the intensity of a band corresponding to the size of Srsf1, demonstrating that Srsf1 directly binds to the E 13A-3 sequence element. When using the E 13A-3mut probe, the binding slightly decreased but was not fully abolished, which is consistent with the presence of additional Srsf1 binding sites in this exon (Supplementary Figure 4B).

### The relative levels of Srsf1 and Elavl1 directly impact the outcome of TrkC alternative splicing

**Figure 4.**
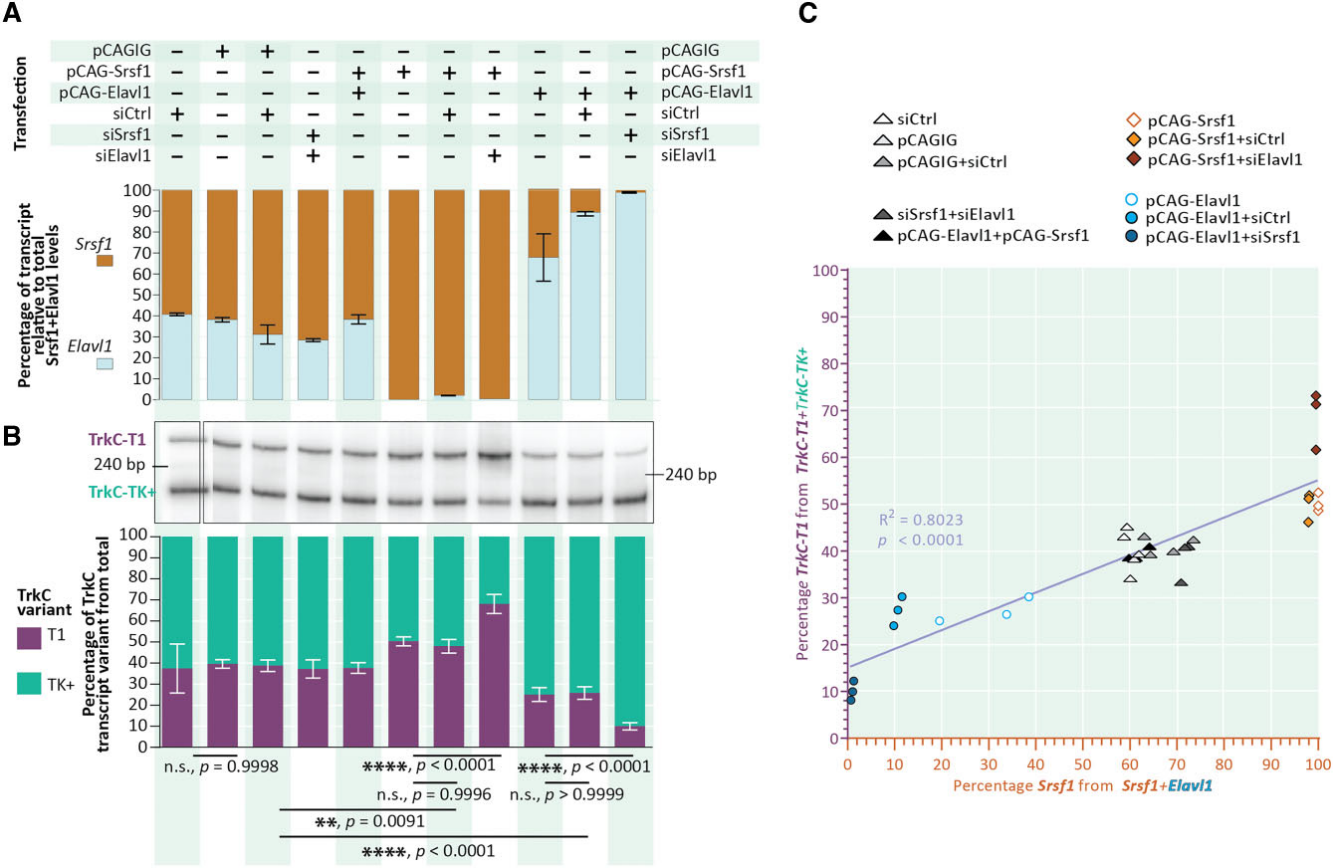
The ratio of Srsf1 to Elavl1 steers the alternative splicing choice between TrkCT1 and TrkC-TK+. (**A**) Srsf1 to Elavl1 ratios were modulated in N2a cells by transfection with either expression constructs, siRNAs against the two transcripts, or combinations thereof. Combinations are indicated above. The resulting ratio of Srsf1 to Elavl1 transcripts was determined by RT-qPCR and plotted below. The percentage of each transcript was calculated based on the C_T_ values by assuming one cycle difference in C_T_ to indicate a twofold difference. The bars are divided at the mean percentage from *N* = 3 replicates. Error bars represent the standard deviation of the three results. The ΔΔC_T_ values for *Srsf1* and *Elavl1* transcript levels relative to *Hprt* transcript levels and matching control sample are summarized in Supplementary Figure 5. (**B**) The effect of the Srsf1 and Elavl1 modulations in (A) was assessed by radioactive, splicing-sensitive PCR specific to the TrkC-T1/TK+ alternative splicing event, as described in Figure 2B. Each lane corresponds to the Srsf1/Elavl1 ratio and transfection conditions indicated above it in panel (A). The quantification was performed by normalizing the intensity of the TrkC-T1 band to the total signal from the TrkC-T1 and TrkC-TK+ bands. The bars are divided at the mean percentage from *N* = 3 replicates. Error bars represent the standard deviation from the mean of the three results. *P* values derived from ordinary one-way ANOVA with Šidak's *post-hoc* multiple comparisons test. (**C**) Correlation analysis of the percentage of Srsf1 in Srsf1 + Elavl1 transcripts with the percentage of TrkC-T1 in the TrkC-T1 + TrkC-TK+ transcripts for the experiment presented in (A) and (B). Dots represent individual biological replicates. pCAGIG – pCAG-IRES-GFP, empty vector; pCAG-Srsf1 – overexpression vector containing the Srsf1 CDS; pCAG-Elavl1 – overexpression vector containing the Elavl1 CDS; siCtrl – control siRNA (siAllstar); siSrsf1 – pool of siRNAs against mouse Srsf1; siElavl1 – pool of siRNAs against mouse Elavl1.

Up to this point, we had shown that Srsf1 and Elavl1 each have a significant impact on TrkC AS, with Srsf1 promoting the formation of TrkC-T1 and Elavl1 that of TrkC-TK+ (Figure 2). We sought to explore whether the ratio between the levels of Srsf1 and those of Elavl1 directly drives the outcome of this AS event. To do so, we transfected combinations of constructs aiming to simultaneously change the levels of Srsf1 and of Elavl1 in N2a cells (Figure 4A). We either upregulated the levels of both RBPs (pCAG-Srsf1 + pCAG-Elavl1), knocked both of them down (si*Srsf1* + si*Elavl1*), or upregulated one while downregulating the other (pCAG-Srsf1 + si*Elavl1* and pCAG-Elavl1 + si*Srsf1*), and then assessed TrkC AS outcomes by splicing-sensitive RT-PCR (see Supplementary Figure 5 for relative quantification of Srsf1 and Elavl1 transcript levels in relation to matching control samples). We first observed that, in all of the control samples (pCAGIG, siCtrl, pCAGIG + siCtrl), *Srsf1* levels and *Elavl1* transcripts were distributed in an about 60%-65% to 35–40% ratio in the total Srsf1 + Elavl1 transcript pool. Increasingly large alterations of the Srsf1 to Elavl1 ratio led to changes in the ratio of TrkC-T1 to TrkC-TK+ that were in line with our previous observations (Figure 4B). When Srsf1 dominated the Srsf1 + Elavl1 ratio (pCAG-Srsf1, pCAG-Srsf1 + siCtrl and pCAG-Srsf1 + siElavl1), the TrkC AS outcome also increasingly shifted towards an increase in TrkC-T1, reaching proportions as high as around 70% from the total TrkC transcript pool. In contrast, when Elavl1 dominated the Srsf1 + Elavl1 transcript pool (pCAG-Elavl1, pCAG-Elavl1 + siCtrl, pCAG-Elavl1 + siSrsf1), the production of TrkC-T1 was suppressed down to levels as low as 10% from the total TrkC transcript pool. Overall, the proportion of TrkC-T1 from the total TrkC transcript pool significantly correlated with the Srsf1-to-Elavl1 ratio (Figure 4C, R^2^ for Pearson goodness of fit of linear regression: 0.8203, *P* < 0.0001).

### Srsf1 and Elavl1 have different expression patterns during cortical neuronal differentiation

The ratio of TrkC-T1 to TrkC-TK+ significantly differs between cortical NPCs and neurons (Figure 1). Splicing factors frequently operate in a combinatorial fashion, with levels that differ between cell types and developmental stages (12,13). Thus, we tested whether the difference between T1 to TK+ levels may result from a cell type-specific divergence of Srsf1 and Elavl1 levels. We performed comparative RNA *in situ* hybridization for *Srsf1* and *Elavl1* on cortical sections from different developmental stages (Figure 4A and Supplementary Figure 4). We detected a strong signal for *Srsf1* in the stem cell compartments of the developing cortex (ventricular zone and subventricular zone, short: VZ and SVZ), while levels in the differentiating neurons of the intermediate zone (IZ) and the cortical plate were significantly lower. This held true across the stages of CFuPN production (E 12.5–E 14.5 (30)), in which TrkC-T1 levels are also strongly elevated in NPCs (26). In contrast, Elavl1 transcripts were distributed more uniformly across the neocortex. Both mRNA expression patterns were maintained up to E 16.5 (Supplementary Figure 6A). Furthermore, we could replicate these results for both *Srsf1* and *Elavl1* by RT-qPCR in prominin-1-sorted E 12.5 NPCs and neurons. Here, too, the levels of *Srsf1* differed strongly and significantly between Prom-1-positive and Prom-1-negative cells, whereas those of *Elavl1* did not (Supplementary Figure 6B). Additionally, an analysis of *Srsf1* and *Elavl1* levels during neurodifferentiation of mouse embryonic stem cells (Supplementary Figure 6C) shows *Srsf1* levels decreasing strongly after the start of neurogenesis, whereas *Elavl1* levels do so at a much milder rate, effectively resulting in the change from a strongly *Srsf1*-dominated transcript pool (day 0, high *Pax6* expression) to one where its levels are reduced fourfold and are thereby closer to those of *Elavl1* (after day 16, low to no *Pax6* expression, high *Rbfox3* expression).

**Figure 5.**
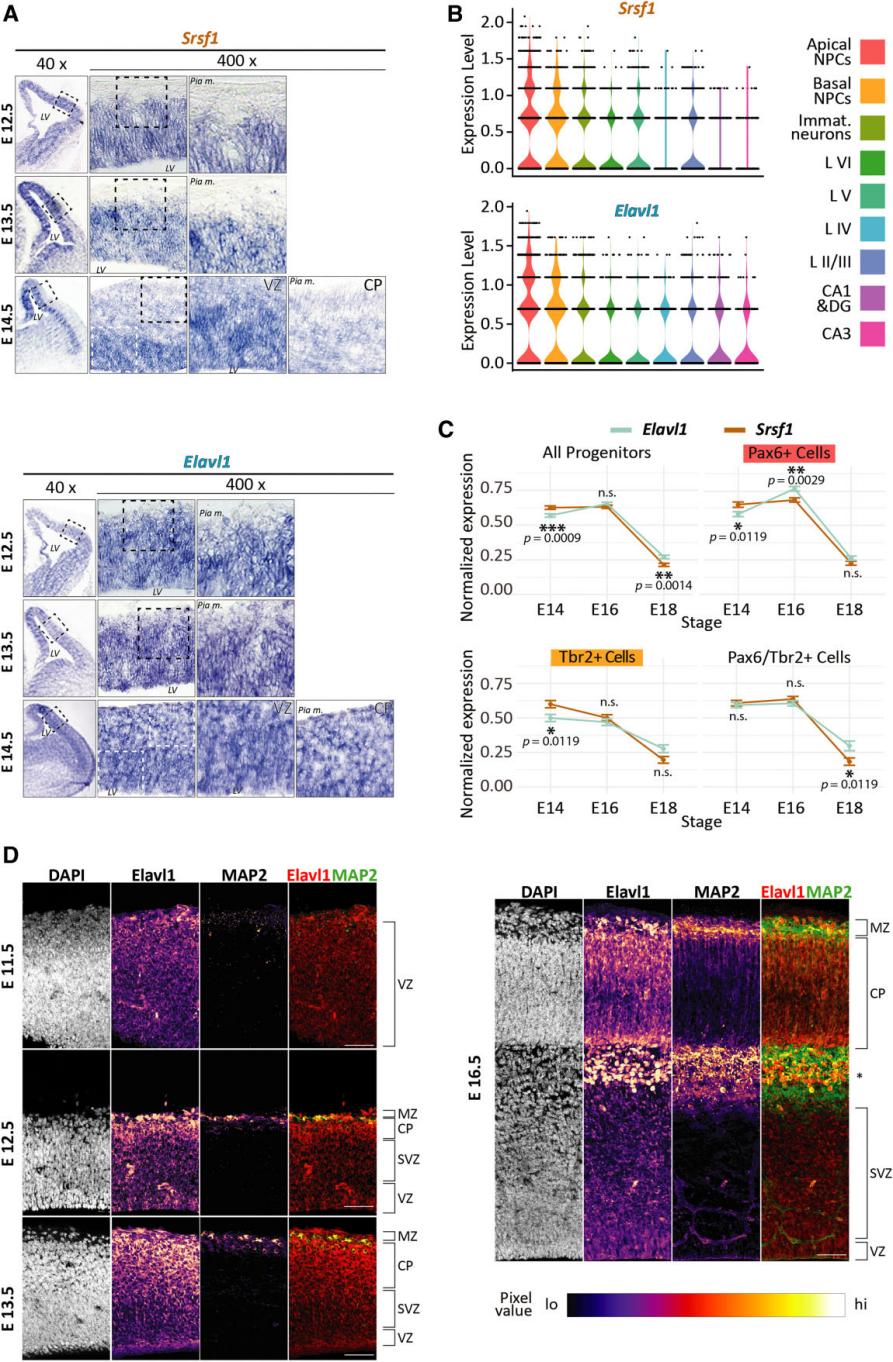
Srsf1 and Elavl1 are differentially expressed in the developing neocortex. (**A**) Srsf1 mRNA levels are high in the ventricular zone and low in the intermediate zone and cortical plate, while Elavl1 is uniformly expressed. RNA *in situ* hybridization with probes against *Srsf1* and *Elavl1* showed different expression patterns in the developing cortex. Exemplary coronal sections of brains at the indicated embryonic stages show strong expression of *Srsf1* in the ventricular zone, with much weaker expression outside this compartment. VZ – ventricular zone, CP – cortical plate, Pia m. – *Pia mater*, LV – lateral ventricle. Dashed boxes represent areas shown magnified in the last or last two magnification insets, respectively. (**B**) *Srsf1* and *Elavl1* are strongly co-expressed in neural progenitor cells in the developing neocortex. Expression plots generated using Seurat v4 and FindAllMarkers package for cells known to be part of the pyramidal neuron lineage. Expression levels generated by the SCTransform package. L – layer. NPCs – neural progenitor cells. Immat. neurons - immature neurons. CA1, CA3 - *Cornu ammonis* areas of the hippocampus; DG – dentate gyrus of the hippocampus. (**C**) The ratio of Srsf1 and Elavl1 mRNA levels in neural progenitor cells flips during corticogenesis. Data obtained from the same analysis as in (B), depicting the stage-specific expression of Srsf1 and Elavl1 mRNAs in different neural progenitor subsets. Asterisks denote significant differences between *Elavl1* and *Srsf1* expression values when testing the fit with a negative binomial model with stage and cell type as interaction terms. Underlying coefficients and further analyses are detailed in Supplementary Figure 6D–F. *Pax6*+ cells: apical NPCs. *Tbr2*+ cells: basal NPCs. (**D**) Elavl1 protein distribution differs from the distribution of its mRNA in the developing cortex. Immunofluorescent micrographs of E 11.5 to E 16.5 cortex sections stained with antibodies against Elavl1 and the neuronal marker MAP2 show an increased signal intensity for Elavl1 in the nascent and formed cortical plate as compared to the VZ/nascent SVZ as in the CP. VZ - ventricular zone, SVZ – subventricular zone, IZ – intermediate zone, CP - cortical plate, MZ – marginal zone. Asterisk denotes putative interneurons, which migrate into the neocortex beginning with E 15.5. Scale bars: 50 μm.

We also used publicly available single cell sequencing data from E 14, E 16 and E 18 mouse cortex (provided by (58)) in order to estimate cell-specific expression levels of both the Srsf1 and Elavl1 mRNAs. We could confirm that *Srsf1* and *Elavl1* are more highly co-expressed in progenitors (Figure 5B). Using the same data, we could observe that, on average, *Elavl1* expression increases in late (E 16) *Pax6*+ progenitors, whereas *Srsf1* expression stays relatively stable until dropping at E 18 (Figure 5C). Similarly, in *Tbr2*+ cells, *Srsf1* expression decreases more precipitously from E 14 to E 18 than the expression of *Elavl1* does. In order to test if *Srsf1* and *Elavl1* expression differs significantly across stages in cortical progenitors, we asked first if the relationship between *Srsf1* and *Elavl1* co-expression is different between stage and cell type, and second, for each combination of stage and cell type, if the distribution of expression values of *Srsf1* differs significantly from that of *Elavl1*. For the first question, we fit a negative binomial model of *Srsf1* and *Elavl1* expression values with ‘stage’ and ‘celltype’ as interaction terms to understand whether *Srsf1* levels depend on *Elavl1* levels. At E 16, *Srsf1* and *Elavl1* showed a significant interaction (Supplementary Figure 6D, StageMouse E16 adjusted *p* = 2.97E-07) compared to the reference E 14 stage, while at E 18 they did not. In other words, *Elavl1* and *Srsf1* expression are concordant at E 16 while at E 14 and E 18 they are not. Similarly, when we looked at the paired mean rank expression of *Srsf1* and *Elavl1* at each stage (Figure 5C), we found that their distributions were significantly different in all progenitors at E 14, (adjusted *P* < 0.001) and E 18 (adjusted *P* < 0.01), and also specifically in *Pax6*-positive progenitors at E 14.

Surprisingly, however, when investigating Elavl1 protein levels in the developing cortex, we observed a discrepancy between the distributions of its transcript and its protein product (Figure 5D). The Elavl1 protein is indeed expressed at constant levels across the germinal zones at E 11.5, and similarly in this cortical zone at later developmental stages. However, from E 12.5 on, the signal observed for Elavl1 in the cortical plate is vastly stronger than that observed in the VZ/SVZ. This pattern is upheld at least up to E 16.5 and indicates that Elavl1 levels in the developing neocortex are primarily regulated on the posttranscriptional level.

Altogether, these data are consistent with the idea that the cell type-specific Srsf1 to Elavl1 ratio in progenitors and neurons changes as differentiation proceeds and, in turn, controls the differing TrkC alternative isoform distribution in these cells.

### Both Srsf1 and Elavl1 control the cell fate in the developing cortex

Given the key role of TrkC-T1 levels in the CFuPN-CPN fate choice in the developing cortex (26) and since Srsf1 and Elavl1 altered the T1 to TK+ ratio (Figure 2), we tested if changing the expression levels of these splicing factors *in vivo* might change the proportion of these cell types in the neocortex. We assessed this by *in utero* electroporating (described in (28)) either overexpression or knockdown constructs for Srsf1 and Elavl1 in the cortical NPCs at E 12.5. Four days post-electroporation, at E 16.5, the cortices were analyzed for the proportion of CFuPNs and CPNs (Figure 5). All constructs co-express GFP, which enables the identification of the progeny of the electroporated NPCs using immunofluorescence. Ctip2 and Satb2 are key determinants of the CFuPN and the CPN fate, and are routinely used to quantify the proportion of these cell types among electroporated (GFP-positive) cells (49–53). We compared the percentages of each category of double positive cells (Ctip2^+^GFP^+^ and Satb2^+^GFP^+^) to the corresponding category in littermate control embryos, which were electroporated with the empty vector (pCAGIG) or a scrambled shRNA. We found that increasing the levels of Srsf1 increased the proportion of GFP-Ctip2 double positive cells at the expense of GFP-Satb2 positive ones, whereas the knockdown of Srsf1 had the opposite effect (Figure 5A). Modulating the levels of Elavl1 in NPCs had the opposite effects: an increase in Elavl1 decreased the proportion of Ctip2-positive cellular progeny and increased that of Satb2-positive daughter cells, and vice versa for the knockdown of Elavl1 (Figure 5B). Hence, both Srsf1 and Elavl1 influence the CFuPN-CPN neuron subtype fate choice in opposing ways.

### The cell fate effects of Srsf1 and Elavl1 are mediated by TrkC-T1

Next, we inquired whether the effects on neuron subtype fate observed for the Srsf1 and Elavl1 level alterations are mediated by TrkC alternative splicing *in vivo*. To address this question, we co-electroporated Srsf1 or Elavl1 expression constructs with constructs aimed at compensating for the increase, respectively decrease, of TrkC-T1 levels caused by these factors and assessed the resulting proportions of Ctip2- and Satb2-positive progeny. Combining the splicing factor expression with a modulation of TrkC-T1 levels led to a mitigation of the fate choice phenotypes observed when solely altering Srsf1 or Elavl1 levels (Figure 5C). The proportions of GFP-Ctip2 and GFP-Satb2 double positive progeny were not significantly different from those in the littermate controls. Altogether, we present evidence that two splicing factors, Srsf1 and Elavl1, regulate TrkC alternative splicing in a cell type-specific manner, which then contributes to control differentiation of NPCs into functionally different neurons.

## DISCUSSION

Developmental fate choices in the production of projection neuron subtypes are crucial for generating both the connections within the neocortex and the ones between the neocortex and other brain structures (30,54). The past years have brought about extensive research on how this species- and region-specific organization of neuron subtypes is achieved during corticogenesis, but the contributing progenitor-intrinsic molecular mechanisms are still incompletely understood. Numerous studies have either identified individual epigenetic or transcription factors that shape the cortical NPC fate (reviewed in (30)) or strived to capture the diversity of embryonic NPC subtypes at the whole-transcriptome level (55–59). Still, there has been little research on how the regulation of RNA processing ensures the levels of fate-determining factors in NPCs that ultimately dictate the fate of their neuronal progeny. In this study, we elucidated an alternative splicing-based mechanism that upholds appropriate levels of TrkC-T1, an isoform of the neurotrophin-3 receptor TrkC, which we have previously shown to be a deep layer (CFuPN) neuron subtype determinant (26). We found that the balance between TrkC-T1 and TrkC-TK+ is stage- and cell type-specific in the developing cortex, and that this balance is controlled antagonistically by the RBPs Srsf1 and Elavl1. Furthermore, we showed that Srsf1 and Elavl1 exhibit differential expression in different cell types in the developing neocortex. Finally, we present direct *in vivo* evidence that these two splicing factors steer the CFuPN-CPN fate choice during corticogenesis. To our knowledge, this is the first example of alternative splicing regulation that controls the ratio between CFuPN and CPN numbers.

### The precise cellular ratio between TrkC-T1 and TrkC-TK+ depends on the cortical cell type

The ratio between TrkC-T1 and TrkC-TK+ has been shown to be crucial for dorsal root ganglion-derived neuron axonogenesis, with the two receptors having dose-dependent antagonistic effects on the number of processes formed (60). In a previous publication (26), we showed that TrkC-T1 impacts signal transduction by sequestering the scaffolding adapter molecule ShcA, and this is not observed with the TrkC-TK+, as had been previously suggested (61,62). In the current study, we also detected TrkC-TK+ in sorted cortical NPCs at E13.5 by RT-qPCR (Figure 1C–E), which is in agreement with findings from a previous study (63). Additionally, this study found that NPCs are responsive to NT-3 signals mediated by TrkC-TK+. Furthermore, in an earlier publication (64), we found that NT-3 production by postmitotic cortical neurons is a key feedback mechanism for the NPCs switching from deep to superficial layer neuron production. Taken together, these results argue for the need for a balance between TrkC-TK+ and TrkC-T1 signaling in cell fate decisions in the NPCs. We indeed found that the ratio of T1 to TK+ shifts in favor of TK+ during cortex development from a whole-tissue perspective (Figure 1B and (26)), and that this is likely due to the change in cellular composition. We found that NPCs and differentiating neurons exhibit different ratios of TrkC-T1 to TK+ (Figure 1E). While we cannot exclude the effect of other cell types, the change in cell type prevalence in the developing cortex from an NPC-dominated tissue (E 12.5) is overwhelmingly driven by the production of postmitotic neurons. This may explain the balance shift observed in bulk tissue.

Previous research indicates that the NPC population at any one cortical development stage may not be homogeneous in their potential to produce differently fated progeny (65), and recent single-cell RNA sequencing studies support this hypothesis (56,58). Given the considerable increase in, for instance, intermediate progenitor numbers over the stages in which TrkC-T1 levels drop in the ventricular/subventricular zone, it may be that, at early cortex development stages, some NPCs exhibit a CFuPN-favoring T1/TK+ balance and others a CPN-favoring one. Our RNA sequencing data analysis (Figure 5B and C) and our previous findings (26) indicate that a changing ratio of Srsf1 and Elavl1 during differentiation may cause a shift from CFuPN production to the production of later-generated fates, such as the CPN fate. To acquire insight into whether this is truly the case *in vivo* and how this impacts cell fate, highly sensitive mass spectrometry techniques, single-cell proteomics and single-cell RNA sequencing at isoform resolution (66,67) on sorted NPCs may provide answers.

### Srsf1 and Elavl1 co-regulate the balance of TrkC-T1 to TrkC-TK+ and steer the CFuPN-CPN fate choice

The work presented here and that of others (60,68) demonstrates the importance of upholding a finely tuned cellular balance between TrkC-T1 and TrkC-TK+. However, until now, we have had no knowledge on the mechanisms regulating this balance. Here, we show by radioactive splicing-sensitive RT-PCR, RT-qPCR, and Western blotting that the splicing factors Srsf1 and Elavl1 have antagonistic effects on the alternative splicing of the TrkC pre-mRNA, with Srsf1 favoring the formation of TrkC-T1 and Elavl1 that of TrkC-TK+ (Figure 2C–H). Additionally, we show that the precise cellular ratio of Srsf1 to Elavl1 transcripts is sufficient for driving the changes observed for TrkC AS (Figure 4).

Previous large-scale RNA sequencing projects and bioinformatic analyses showed that alternatively spliced last exons are an especially finely regulated class of alternative splicing events in developing neural cells. Their alternative inclusion in mRNA often leads to the expression of two main protein isoforms with distinct C-terminal protein domains that frequently undergo signaling-relevant phosphorylation (19,69). Similarly, alternative splicing of penultimate exons whose exclusion induces a frameshift, leading to proteins with altered C-termini, and is highly regulated during neuronal differentiation (70). Our findings regarding TrkC alternative splicing and stability regulation fit these patterns. Unexpectedly, though, in this instance, the counterplayer of the regulatory SR protein (Srsf1) is not an hnRNP protein, as is frequently the case (71–73), but Elavl1. Elavl1, also known as HuR, has been more commonly associated with mRNA stability and translational regulation rather than alternative splicing. It binds to AU-rich elements in the 3′ UTR of mRNAs and thereby stabilizes them. Nonetheless, Elavl1 has also been associated with the regulation of alternative splicing in some cases (43,74–78). Intriguingly, we see a potential dual role of Elavl1, acting in the splicing choice between the TrkC-T1 and TrkC-TK+ transcript, but also in the differential stabilization of the two (Supplementary Figure 4F). This may be due to the presence of several strong predicted Elavl1 binding sites in the 3′ UTR of TrkC-T1 (Supplementary Figure 4E), whereas no such binding sites could be detected in the 3′ UTR of TrkC-TK+. In contrast, the role of Srsf1 in TrkC AS clearly depends on a splicing enhancer in the last third of exon 13A (E 13A-3), which is crucial for TrkC-T1 formation (Figure 3H–K) and loses some of its enhancing ability when Srsf1 is knocked down (Figure 3F–G).

Based on these results and together with the results of the correlation analysis of the Srsf1/Elavl1 ratio with TrkC-T1/TrkC-TK+ (Figure 4), we hypothesize that, for TrkC-T1 to be formed, Srsf1 levels have to be considerably higher than Elavl1 levels. If the role of Elavl1 in alternative splicing is overridden by Srsf1, then, TrkC-T1 can result from this processing step. As a fine-tuning regulatory step, Elavl1 can then bind to the 3′ UTR of the processed TrkC-T1 mRNA, having a modest stabilizing effect. Due to the small effect size of the Elavl1 knockdown on TrkC-T1 levels in the actinomycin D-treated cells (around 10% change in PSI compared to the DMSO + siElavl1 sample) versus the much larger effect on alternative splicing (around 50% change in PSI compared to the siControl, Figure 2D–E and Supplementary Figure 2A), we suggest that Elavl1 primarily acts as a splicing regulator to balance the TrkC-T1 and TrkC-TK+ isoforms. Taken together, these findings suggest a regulatory network that fine-tunes TrkC transcript variant levels, orchestrated by Srsf1 and Elavl1.

**Figure 6.**
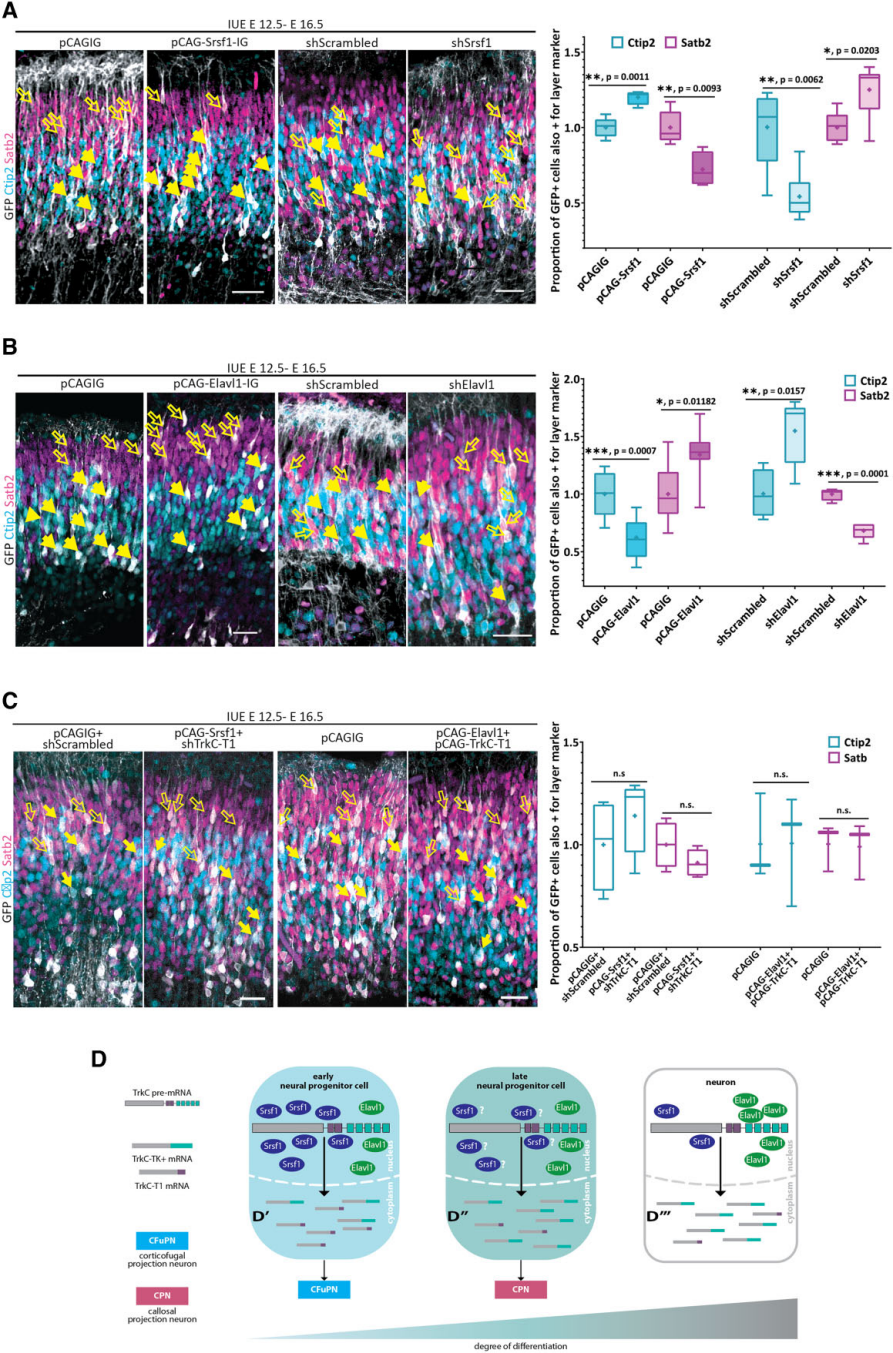
Srsf1 and Elavl1 act antagonistically on TrkC-T1 levels to control the numbers of corticofugal neurons (CFuPNs) and callosal projection neurons (CPNs) in the developing cortex. (**A**) Srsf1 overexpression increases the number of CFuPNs *in vivo*,while its downregulation decreases it, and elicits the opposite effect on CPNs. Srsf1 overexpression constructs (pCAG-Srsf1-IRES-GFP) or empty vector constructs (pCAG-IRES-GFP, abbreviated pCAGIG) were electroporated into the lateral ventricles of E 12.5 embryos. Cells co-expressing GFP and one of the neuronal fate markers Ctip2 (CFuPN subtype) or Satb2 (CPN subtype) were quantified at E 16.5. *N* = 5. Similarly, plasmids expressing either a scrambled shRNA or an shRNA directed against Srsf1 were electroporated into the lateral ventricles of E 12.5 embryos. Brains were analyzed at E 16.5 as described in (A). shScrambled: *N* = 5. shSrsf1: *N* = 6. *P* values derived from unpaired Student's *t* test with Welch's correction. Box plot whiskers: minima and maxima of the sample. Horizontal line: median. Plus sign: mean of the sample. Empty arrows: GFP + Satb2 double-positive cells. Full arrows: GFP + Ctip2 double-positive cells. Scale bars = 50 μm. (**B**) Elavl1 expression level manipulations have an effect opposite to that of Srsf1 on the proportions of CFuPN/CPN neurons *in vivo*. Experiment performed as described in (A), using pCAG-Elavl1-IRES-GFP expression constructs. pCAGIG: *N* = 10. pCAG-Elavl1: *N* = 7. Similar to (A), we also used an shRNA against Elavl1. shScrambled: *N* = 4. shElavl1: *N* = 5. Statistics and labeling as in (A). Scale bars = 50 μm. (**C**) The effects of Elavl1 and Srsf1 on CFuPN-CPN neuron production depend on TrkC-T1 levels. Constructs were electroporated as shown and the quantification was performed as described in (A). The effects of Srsf1 overexpression are abolished upon the knockdown of TrkC-T1. *N* = 4 for pCAGIG + shScrambled, *N* = 5 for pCAG-Srsf1 + shTrkC-T1. Mean ± SD: Ctip2 - 1 ± 0.2237 in control versus 1.141 ± 0.1787 in pCAG-Srsf1 + shTrkC-T1; Satb2 - 1 ± 0.1109 in control versus 0.9914 ± 0.06534 in pCAG-Srsf1 + shTrkC-T1. Similarly, Elavl1 overexpression has no effect on the proportion of Ctip2 or Satb2 cells when TrkC-T1 levels are increased. *N* = 3 for both pCAGIG and pCAG-Elavl1 + pCAG-TrkC-T1. Mean ± SD: Ctip2 - 1 ± 0.2146 in control versus 1.007 ± 0.1723 in pCAG-Elavl1 + pCAG-TrkC-T1; Satb2 - 1 ± 0.1159 in control versus 0.99 ± 0.14 in pCAG-Elavl1 + pCAG-TrkC-T1. Statistics and labeling as in A. Scale bars = 50 μm. (**D**) Proposed model of the neuronal subtype fate regulation by Srsf1 and Elavl1 and TrkC isoform expression in the developing cortex. Srsf1 is labelled with question marks as we have only shown a change in mRNA.

There are only few well-documented cases in which transcript variant ratios are involved in cell fate decisions, and even fewer regarding neuron subtype decisions. One known case of a splicing factor being involved in a neuron subtype decision in corticogenesis is that of SRRM4 (79). This study showed that SRRM4 impacts the numbers of Tbr1- and Satb2-positive neurons, but it does not show a regulation of the overarching CFuPN fate. Tbr1-positive (corticothalamic projection neurons, a subset of CFuPNs) and Satb2-positive neurons (CPNs) only show minor overlap in their generation time frames and minimal shared layer occupancy (30), which is why the ultimate impact magnitude of this fate control mechanism is unknown. Furthermore, the alternatively spliced transcripts mediating this function of SRRM4 were not described (79). Here, we show, for the first time, that the splicing factors Srsf1 and Elavl1 drive significant changes in the fate acquisition process for CFuPN and CPN in the developing cortex (Figure 6), an effect mediated by their antagonistic effects on TrkC alternative splicing and stability and the resulting balance between the receptor isoforms TrkC-T1 and TrkC-TK+ (Figure 2F–G, Supplementary Figures 4F and 6A).

### Srsf1 and Elavl1 levels define cell-type specific splicing-regulatory environments in the developing cortex

Across the adult mammalian tissues investigated previously, it has been found that AS frequency is highest in the brain, a phenomenon likely caused by the high number of RBPs expressed in this tissue and their dynamic and variable interaction networks (22,80). A previous study has emphasized the impact of alternative splicing on cortex development, as a major tissue-wide splicing switch occurs prenatally, at E 14.5 (81). Here, we show how two novel players in alternative splicing regulation during neurodevelopment, Srsf1 and Elavl1, steer fate choices early in cortex development. Using RNA *in situ* hybridization, immunofluorescence, and RT-qPCR on sorted cortical aRGCs and postmitotic neurons, we could show that Srsf1 and Elavl1 expression levels define TrkC alternative splicing-regulatory environments in the developing cortex that are distinct between progenitors and neurons (Figure 5 and Supplementary Figure 6). Although our analysis was focused on TrkC alternative splicing, the balance of Srsf1 and Elavl1 likely affects splicing events and mRNA stability for additional targets in a cell type-specific manner. We found Srsf1 expression to contrast starkly between the two cell types, with far stronger expression in aRGs than neurons, while Elavl1 mRNA levels were more similar across the cortical tissue (Figure 5A) and the two cell types (Supplementary Figure 6B and C).

Unexpectedly, the Elavl1 protein distribution does not entirely follow the distribution of its transcript (Figure 5B), accumulating more strongly in the cortical plate of the developing neocortex (Figure 5D). This finding points to a ratio of Srsf1 to Elavl1 in postmitotic neurons that could be much more strongly dominated by Elavl1 than anticipated from the mRNA data. We have recently shown that cortex development is rife with other instances of stark discrepancies between transcript and protein levels in different cell types (82). A good example for this phenomenon is the chromatin-associated CPN fate marker Satb2 (51), whose protein is solely detected in this postmitotic neuron subtype, whereas its transcripts are present without being translated in a much broader spectrum of cortical cells, including in neural progenitor cells (82). Our data indicate that Elavl1 protein and transcripts also exhibit such a discrepancy.

The role of Srsf1 in central nervous system (CNS) development has not been extensively addressed before, likely because the embryonic lethality of Srsf1 deletion mouse lines has been ascribed to cardiovascular and skeletal defects (83,84). As for Elavl1, the only instance of developmental alternative splicing regulation through it and other Elav protein family members has only recently been reported in the fruit fly CNS (85). In mammalian corticogenesis, Elavl1 has solely been shown to act as a stage-specific regulator of mRNA translation in the mouse, exhibiting the same expression pattern we could observe in our work on both RNA and protein level (86). In this earlier publication, Elavl1 was shown to alter the phosphorylation states of core ribosomal components through collaborative action with the eIF2-alpha kinase 4, which impacts the association of transcripts with ribosomal components and the formation of polysomes. While the authors showed changes in *Ctip2* mRNA distribution in the unbound versus 40S–60S and polysomal fractions of Elavl1 conditional knockout animals, this was not directly causally linked to a change in the CFuPN/CPN fate. Our results suggest that Elavl1 participates in the CFuPN/CPN fate decision through alternative splicing and mRNA stability regulation (Figures 3 and 5). Combined, these findings pose the question of whether Elavl1 may have a dual role in establishing the CFuPN fate, both via regulating the alternative splicing of TrkC in NPCs and by Ctip2 translation control after cell cycle exit in deep layer neurons. Further studies employing a Dcx-promoter-driven knockdown or overexpression of Elavl1 may help to disentangle the pre- and postmitotic involvement of Elavl1 in the CFuPN fate.

In conclusion, we show for the first time direct *in vivo* evidence that Elavl1 and Srsf1 contribute to the fate switch between CFuPN and CPN in the developing cortex, acting at the level of TrkC splicing and stability. Since the cell type-specific distribution of Srsf1 and Elavl1 is maintained up to E 16.5 (Supplementary Figure 5), outside of the time window in which CFuPN and CPN fate acquisition overlap, their balance may participate in other NPC- or neuron-specific splicing events that are independent of TrkC alternative splicing but of importance to later developmental processes, such as the neurogenesis-gliogenesis switch at E 17.5. This avenue remains to be explored using manipulations of Srsf1 and Elavl1 levels at other developmental time points.

## Supplementary Material

gkad703_Supplemental_file

## Data Availability

The data underlying this article are available in the article and in its online supplementary material.
