# Correction to ‘Srsf1 and Elavl1 act antagonistically on neuronal fate choice in the developing neocortex by controlling TrkC receptor isoform expression’

**DOI:** 10.1093/nar/gkad1238

**Published:** 2023-12-24

*Nucleic Acids Research*, Volume 51, Issue 19, 27 October 2023, Pages 10218–10237, https://doi.org/10.1093/nar/gkad703.

In the originally published version of this manuscript, author Ekaterina Epifanova's affiliation information required correction, as the author's affiliations were given as “1, 5”, instead of “1”.

This has been corrected online.

