# Supplementary material for: Srsf1 and Elavl1 act antagonistically on neuronal fate choice in the developing neocortex by controlling TrkC receptor isoform expression
Source: Nucleic Acids Res. 2023 Sep 11;51(19):10218–37. doi: 10.1093/nar/gkad703 (PMC10602877; doi:10.1093/nar/gkad703)
Supplement: gkad703_Supplemental_file [file gkad703_supplemental_file.pdf]

Supplementary figure 1
pertaining to Figure 1

A

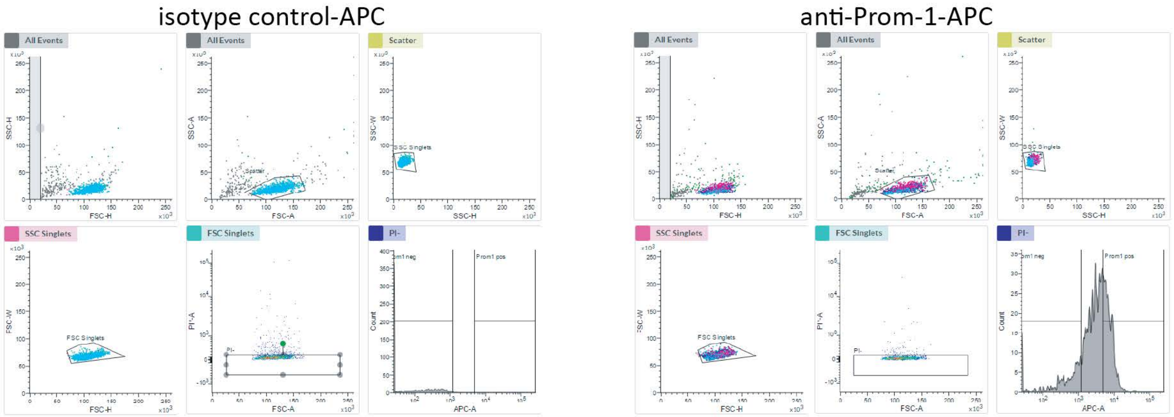

B

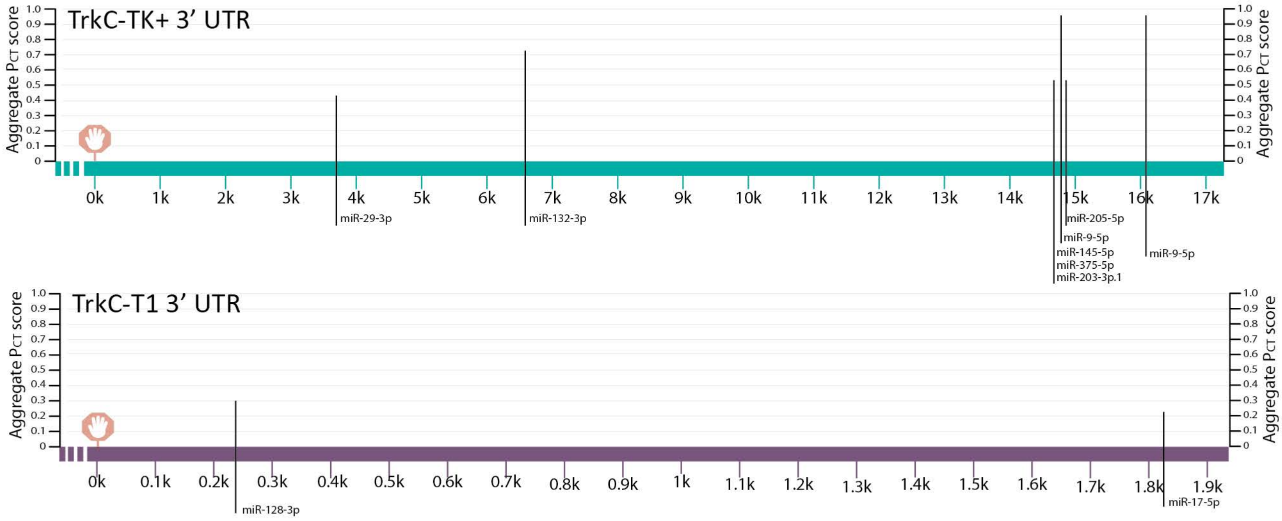

TrkC-TK+-binding miRNA family	Conserved sites total	Conserved 8mer sites	Conserved 7mer-m8 site	Conserved 7mer-1A sites	Poorly conserved sites total	Poorly conserved 8mer sites	Poorly conserved 7mer-m8 site	Poorly conserved 7mer-1A sites	6mers	Aggregate Pct
**miR-9-5p**	2	0	2	0	1	0	0	1	3	0.97
miR-132-3p/212-3p	1	1	0	0	2	0	1	1	5	0.71
miR-205-5p	1	1	0	0	2	0	1	1	5	0.53
miR-145-5p	1	0	1	0	5	0	2	3	3	0.52
miR-29-3p	1	1	0	0	0	0	0	0	5	0.44
miR-208-3p	1	0	0	1	1	0	0	1	2	0.43
miR-375-3p	1	0	0	1	5	0	1	4	3	0.4
miR-24-3p	0	0	0	0	3	0	2	1	0	0.24
miR-143-3p	0	0	0	0	7	1	4	2	7	0.16
miR-203-3p.1	1	0	0	1	4	2	2	0	9	0.12

TrkC-T1-binding miRNA family	Conserved sites total	Conserved 8mer sites	Conserved 7mer-m8 site	Conserved 7mer-1A sites	Poorly conserved sites total	Poorly conserved 8mer sites	Poorly conserved 7mer-m8 site	Poorly conserved 7mer-1A sites	6mers	Aggregate Pct
**miR-128-3p**	1	0	1	0	0	0	0	0	0	0.3
miR-17-5p/20-5p/93-5p/106-5p	1	0	0	1	2	0	1	1	4	0.23
miR-24-3p	0	0	0	0	1	0	0	1	0	0.22
miR-425-5p/489-3p	0	0	0	0	1	0	1	0	1	0.13

Supplementary figure 1

A Details of the FACS gating strategy for the Prom-1-based primary cortical cell sorting presented in Figure 1 C-E.

In brief, cells were separated based on the height and amplitude of side scatter (SSC) and forward scatter (FSC) signals in order to discriminate against cell doublets. From the singlets, only propidium iodide (PI)-negative cells were considered viable and included in the sorting based on the APC signal of either the Prom-1 antibody (plots on right side; Prom-1-positive: magenta, Prom-1-negative: dark blue) or a corresponding isotype control antibody (plots on left-hand side; detected singlets: light blue).

B miRNAs predicted to bind the different 3' UTRs of TrkC-TK+ and TrkC-T1

Transcript sequences were analyzed using the TargetScan algorithm (targetscan.org[35]). Putative miRNA binding sites and aggregate P_{CT} scores are indicated with vertical lines across the length of each 3' UTR, beginning with the stop codon (stop sign). Putative binding miRNA characteristics are summarized in the two tables below.

Supplementary figure 2

A

siRNA against…	%TrkC-T1 from total	SD	% change from siCtrl	Expression in WT N2A	
				Value	Rank
Celf1	26.16	0.78	-1.15	10.8	95
Celf2	25.56	0.64	-3.43	4.5	3
Elavl1	71.55	3.82	76.61	11.5	98
*Elavl2**	47.35	8.90	6.54	9.1	83
Hnrnph2	48.88	0.65	20.66	9.2	84
Hnrnpl	52.11	4.08	28.63	10.6	94
Khdrbs3	40.44	6.94	-0.17	5.0	17
Mbnl1	24.49	0.07	-7.48	10.7	94
Mbnl2	26.10	0.22	-1.41	10.7	95
Mbnl3	26.59	0.25	0.45	4.8	11
Nova1	23.46	1.68	-11.38	9.0	81
Nova2	22.02	0.95	-16.80	7.5	68
Ptbp1	42.28	1.22	4.36	10.9	96
Ptbp2	40.94	3.23	1.06	10.6	94
Rbfox2	25.07	2.36	-5.27	9.4	85
Rbm22	55.11	2.18	36.04	9.3	84
Rbm47	42.24	2.50	4.28	4.6	3
Rbm5	40.26	8.92	-0.63	9.4	85
Srpk1	20.94	0.02	-23.17	10.9	96
Srrm2	26.65	1.65	-2.23	11.1	97
Srsf1	13.14	1.79	-51.80	10.1	91
Srsf10	20.24	2.48	-25.74	11.1	97
Srsf11	25.54	0.39	-6.31	9.5	87
Srsf12	30.35	0.46	11.34	5.1	25
Srsf2	27.09	0.02	-0.64	11.7	99
Srsf3	24.27	2.33	-10.97	9.3	84
Srsf4	26.87	1.07	-1.42	9.3	84
Srsf5	20.80	4.75	-23.71	8.6	78
Srsf6	19.05	0.12	-30.11	9.6	87
Srsf7	24.70	1.97	-9.38	9.0	81
Srsf9	26.11	1.98	-4.22	9.3	84
Tra2a	48.31	2.40	19.26	9.7	88
U2af1	47.38	3.50	16.96	9.0	81
Ctrl	*27.26*	*0.82*	*0.00*	-	-
Hprt	-	-	-	10.5	94
Actb	-	-	-	12.8	100

B

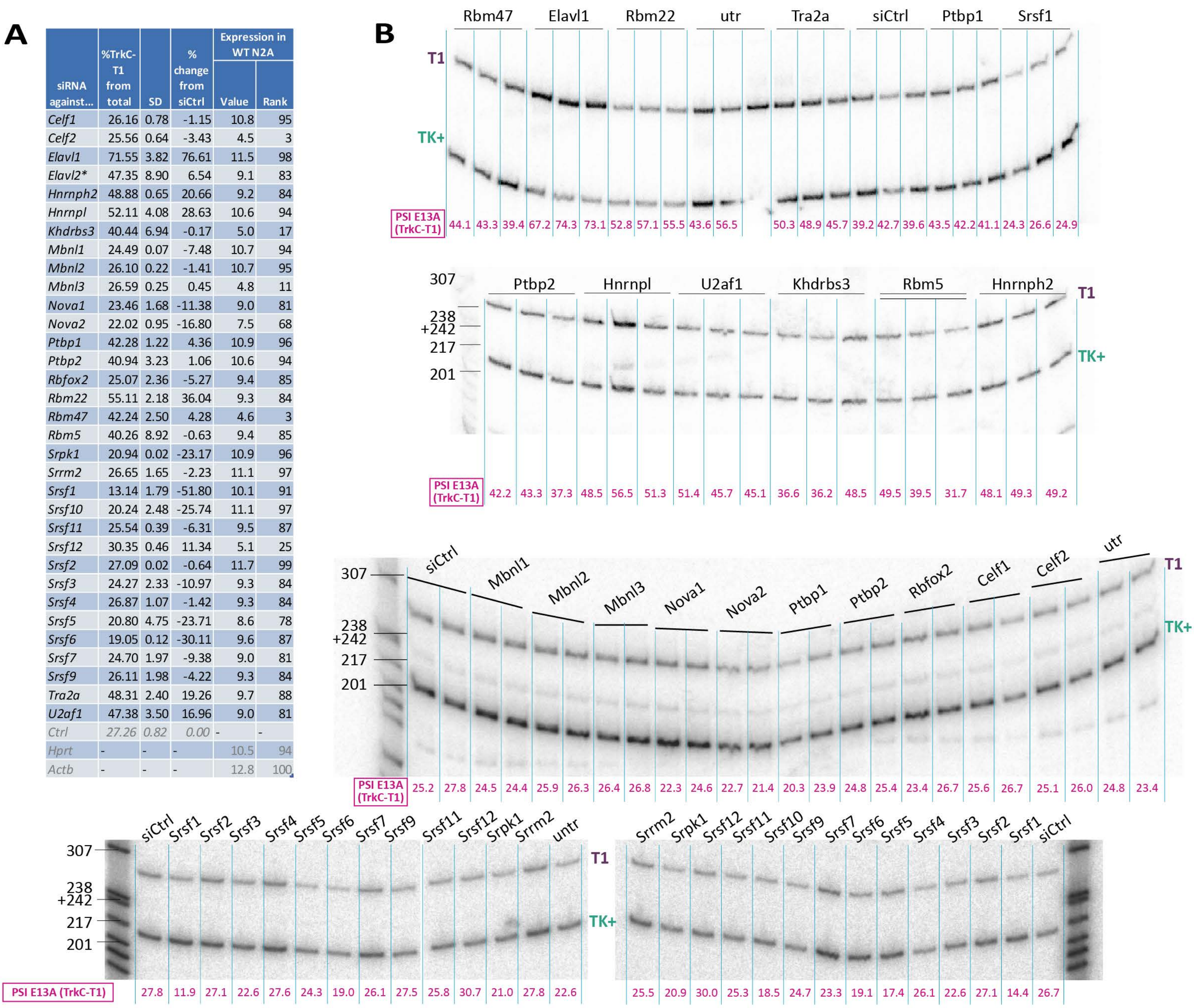

C

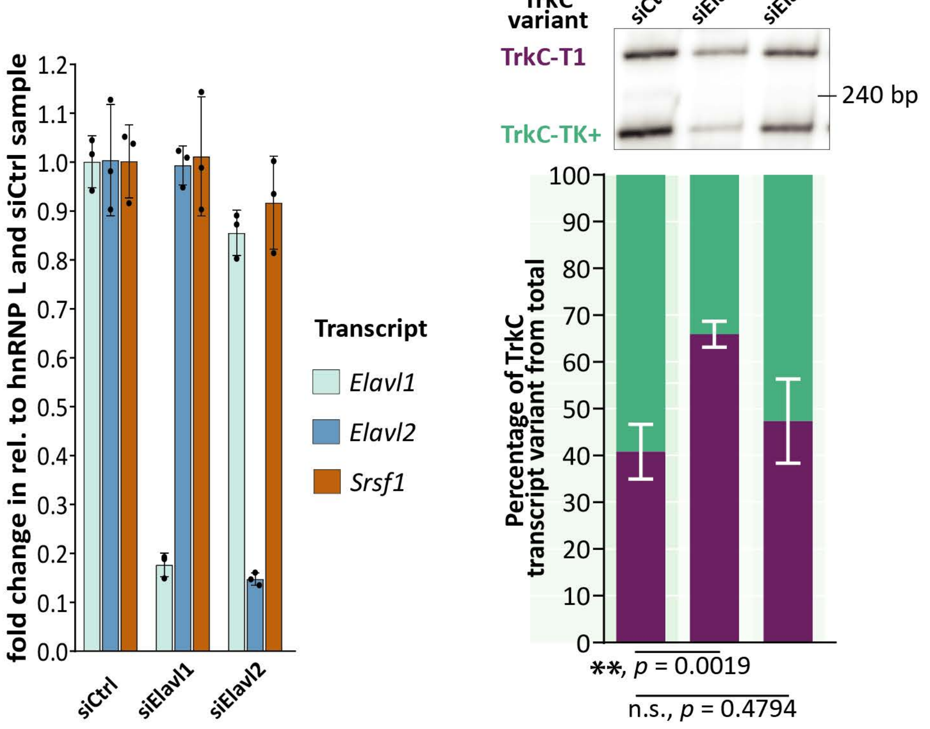

Supplementary figure 2 (pertaining to Figure 2)

A Values of splicing factor knockdown-induced TrkC-T1 changes shown in Figure 2D and their expression values and ranks in N2A cells.

For the % TrkC-T1 from total, values represent the mean of three biological replicates from one siRNA knockdown experiment. The mean PSI difference from control was calculated with respect to the mean percentage of TrkC-T1 in the matching control siRNA samples. The result from an exemplary siCtrl-transfected sample is shown in the table as "Ctrl". Several rounds of experiments were performed, with TrkC-T1 levels slightly differing in untransfected cells, depending on the passage number of the cells. For all of the knockdown samples, the percent change from total was reported with regard to the TrkC-T1 percentage in the control siRNA-transfected sample of that experiment in order to avoid falsification due to the degree of differentiation of N2a cells, which we observed decreases at high passage numbers. * - the effects of knocking down *Elavl2* are described in (C).

For the expression of the tested splicing factors in N2a cells, the values were retrieved from the microarray expression dataset on wild type N2A cells from (87) (GEO dataset GDS5140). *Hprt* and *Actb* are housekeeping gene transcripts included as controls. The rank is a variable reported within the dataset and indicates the percentile of the expression level of the respective SF as compared to all genes in the dataset. *Hprt* and *Actb* are in the 94th and 100th percentile, respectively, placing them amongst the top expressed genes. Values retrieved by the GEO interface.

B Radioactive splicing-sensitive PCR gels showing effects of splicing factor knockdowns on TrkC AS. The gels are shown for all of the tested splicing factors, along with the respective control samples (siControl/siCtrl and untransfected). Although the exact percentage of TrkC-T1 from the total TrkC transcript pool changes slightly due to varying stages of differentiation of the employed N2a cells, Elavl1 and Srsf1 consistently generate the effects reported in Figure 2. The percentage of TrkC-T1 (upper band) resulting from its quantification in relation to total TrkC transcripts (TrkC-T1+TrkC-TK+ band intensities) is indicated below each of the lanes.

C Knocking down other Elavl family members does not impact TrkC AS

(left) Graphs showing the successful knockdown of Elavl1 or Elavl2, their lack of an effect on Srsf1 levels, and the resulting distribution of TrkC-T1 and TrkC-TK+ (right). Gel image above is of an exemplary radioactive splicing-sensitive PCR performed as described in Figure 2 B. N =3; error bars represent standard deviation. P values derived from unpaired Student's t test.

Supplementary figure 3
pertaining to Figure 2

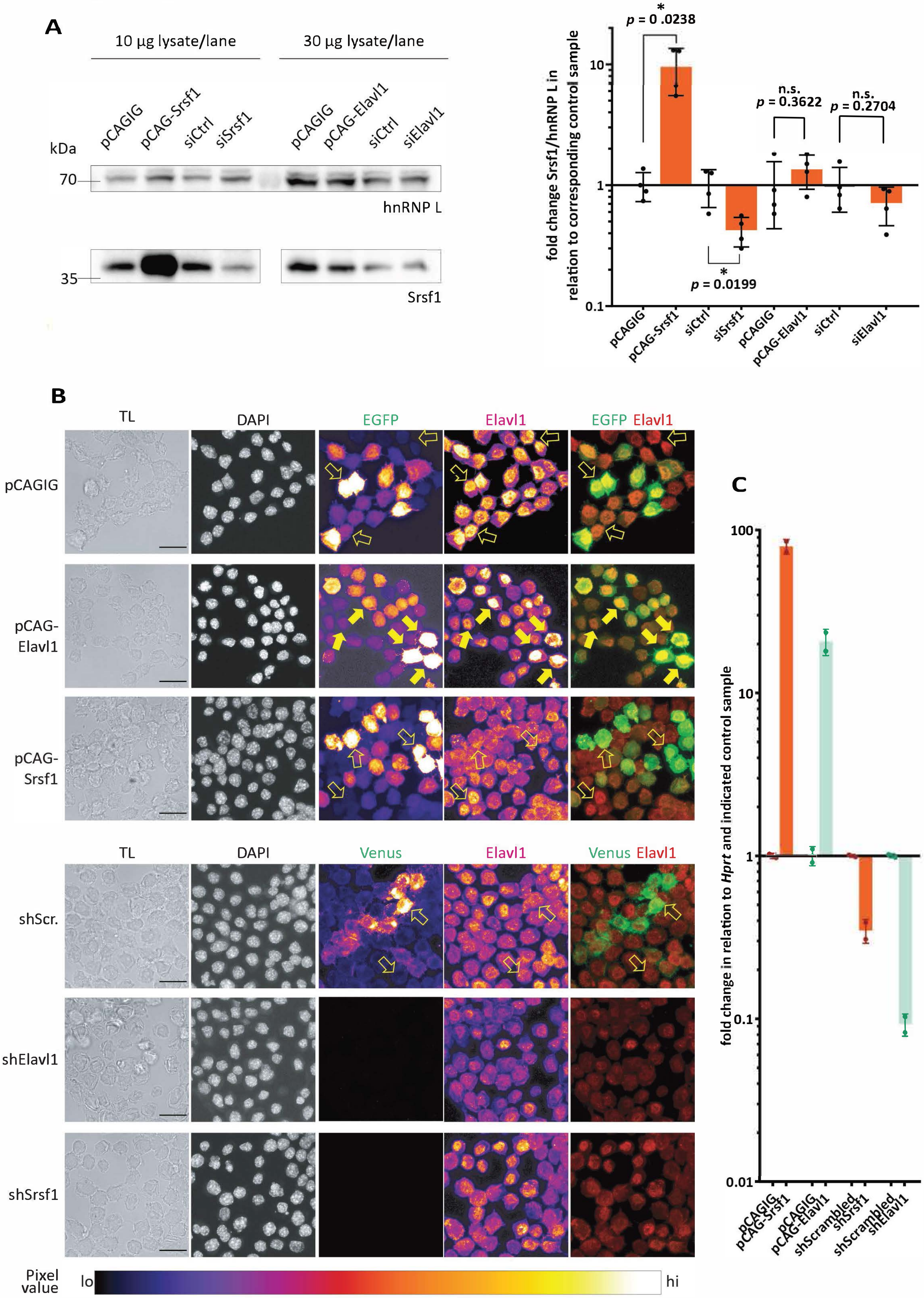

Supplementary figure 3 (pertaining to Figure 2): Elavl1 and Srsf1 do not exhibit cross-regulation.

A The protein levels of Srsf1 are not affected by the overexpression or knockdown of Elavl1. Exemplary Western blotting result showing whole cell lysate proteins in the indicated amounts and generated from N2a cells transfected with the indicated constructs. Quantification is summarized in the graph on the right-hand side. Each overexpression or knockdown sample is compared to its matching control (empty vector pCAGIG or siCtrl). N =4; p values derived from unpaired Student's t test with Welch's correction.

B The levels of Elavl1 are only affected by the Elavl1 overexpression plasmid and the shRNA against Elavl1, but not by the Srsf1 overexpression plasmid or shRNA. Immunofluorescence micrographs depicting N2a cells after transfection with the constructs indicated on the left. EGFP and Venus signals are derived from the fluorophores encoded on the respective plasmids, and act as transfection markers. Elavl1, both endogenous and exogenous, is detected by immunostaining. In the empty vector control (pCAGIG), the shScrambled control, and the Srsf1 overexpression (pCAG-Srsf1) samples, the transfection marker signal does not correlate with changes in Elavl1 levels. Empty arrows indicate some of the mismatches between transfection levels and Elavl1 levels (cells showing large differences in EGFP or Venus levels but comparable levels of Elavl1). In contrast, transfection with the Elavl1 overexpression plasmid leads to a consistent increase in Elavl1 levels, while treatment with the shRNA against Elavl1 decreases Elavl1 levels overall. Full arrows in the Elavl1 overexpression samples (pCAG-Elavl1) indicate some of the matches between transfection level and increases in Elavl1 levels. Cells with a knockdown of Srsf1 (shSrsf1) show Elavl1 levels similar to those in the shScrambled control, whereas cells transfected with an Elavl1 knockdown construct (shElavl1) show reduced overall Elavl1 levels. Scale bar: 20 µm. TL – transmitted light image, shScr – shScrambled. Color LUT: Inferno (ImageJ).

C Expression of *Srsf1* and *Elavl1* relative to *Hprt* levels and the indicated control (to the left of the sample) in duplicate samples derived from the same experiment as the samples imaged in (B), as determined by RT-qPCR. Further information on the knockdown and overexpression efficiency of the constructs can be found in Supplementary figure 5.

Supplementary figure 4
pertaining to Figure 3

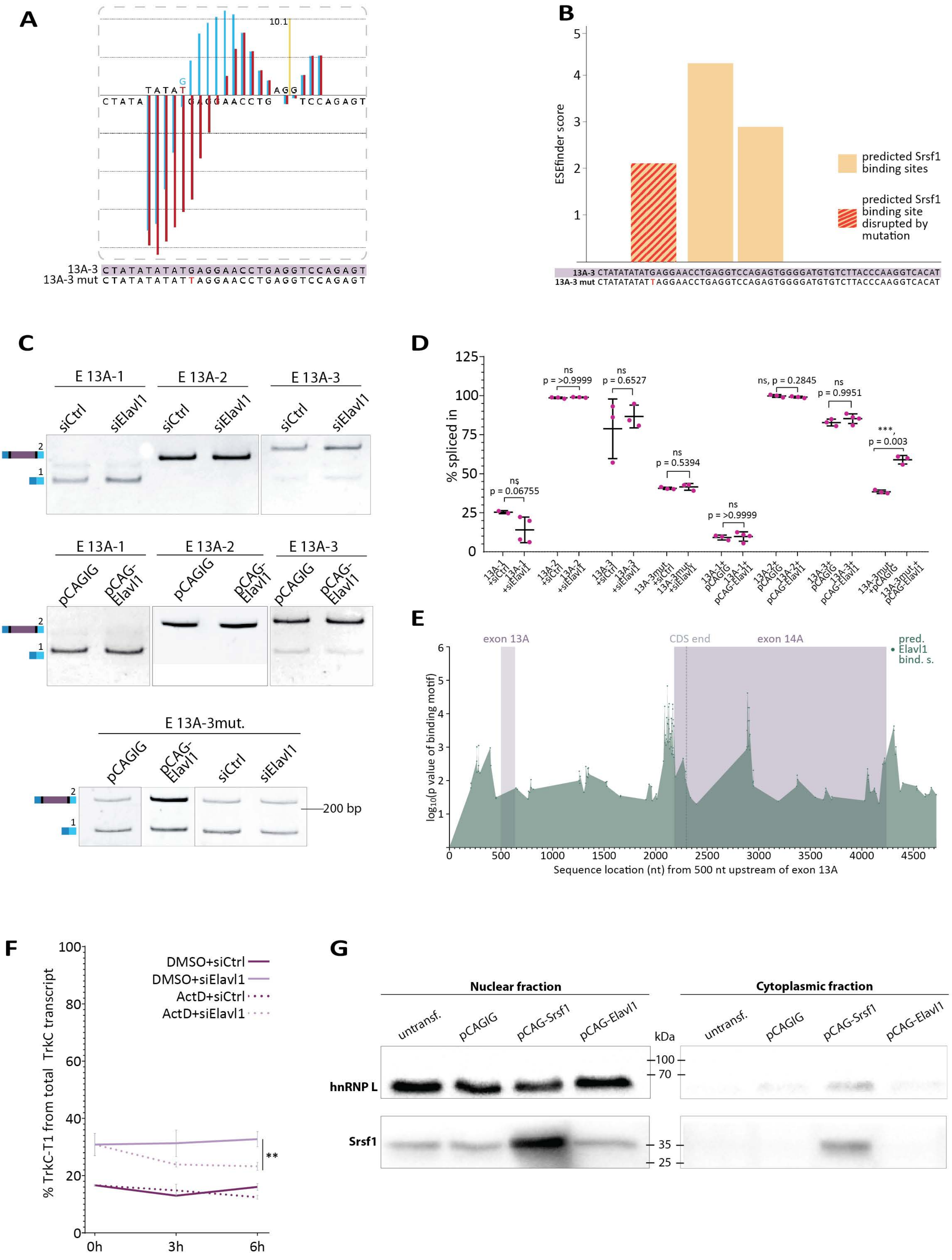

Supplementary figure 4 (pertaining to Figure 3): TrkC transcript levels are regulated by an Srsf1-dependent exonic splicing enhancer element in the first TrkC-T1-specific exon, exon 13A, while Elavl1 controls splicing and stability.

A Fragment 13A-3 contains a sequence stretch predicted by HEXplorer to be crucial for the splicing-enhancing properties of this exon part. A mutation at this site (G->T, indicated in red in lower sequence) is predicted to strongly disrupt its splicing-enhancing properties. HEXplorer profile of the wild type 13A-3 sequence is depicted in blue. Profile of 13A-3 with the disruptive mutation (13A-3 mut) depicted in red.

B The third part of exon 13 (13A-3) was found to contain three high-strength putative Srsf1 binding sites using the ESEfinder tool (48). The first of these sites overlaps the nucleotide predicted by HEXplorer to disrupt the splicing-enhancing properties of fragment 13A (G->T, depicted in red in lower sequence). Analyzing the mutated 13A-3 sequence with the same tool did not predict a functional binding site at this location anymore.

C-F Alteration of Elavl1 levels does not affect the splicing of exon 13A but differentially impacts on the stability of TrkC transcripts.

C and D Neither a knockdown nor an overexpression of Elavl1 affected the behaviour of the splicing reporters containing fragments of exon 13A (quantification in **I**). N=3. P values derived from ordinary ANOVA test with Šidak's post-hoc multiple comparisons test. Overall p value: <0.0001.

E Intron 13 and the 3' UTR of TrkC-T1 in exon 14A both contain several high-probability putative binding sites for Elavl1 (RBPmap(40)).

F The impact of Elavl1 on TrkC transcript stability was assessed by treating N2A cells with actinomycin D (10 µg/mL) and harvesting RNA after the indicated number of hours. Decreasing Elavl1 levels by knockdown led to a shift in the decay rates of TrkC transcripts, resulting in a decreased proportion of TrkC-T1 after six hours of actinomycin D treatment. **, p = 0.0054. N = 3; P value derived from unpaired, two-tailed Student's t test.

G Western blot used to test the efficiency of the nuclear-cytosolic fractionation of Srsf1 expression in N2a cells transfected with either a Srsf1 overexpression construct (pCAG-Srsf1) or the empty vector (pCAGIG). The Srsf1 overexpression sample shows a signal for Srsf1 in the cytosolic fraction, likely brought about by the magnitude of the overexpression (also see Supplementary figure 3 A and Supplementary figure 5 for Srsf1 protein and transcript levels upon overexpression).

Supplementary figure 5
pertaining to Figure 4

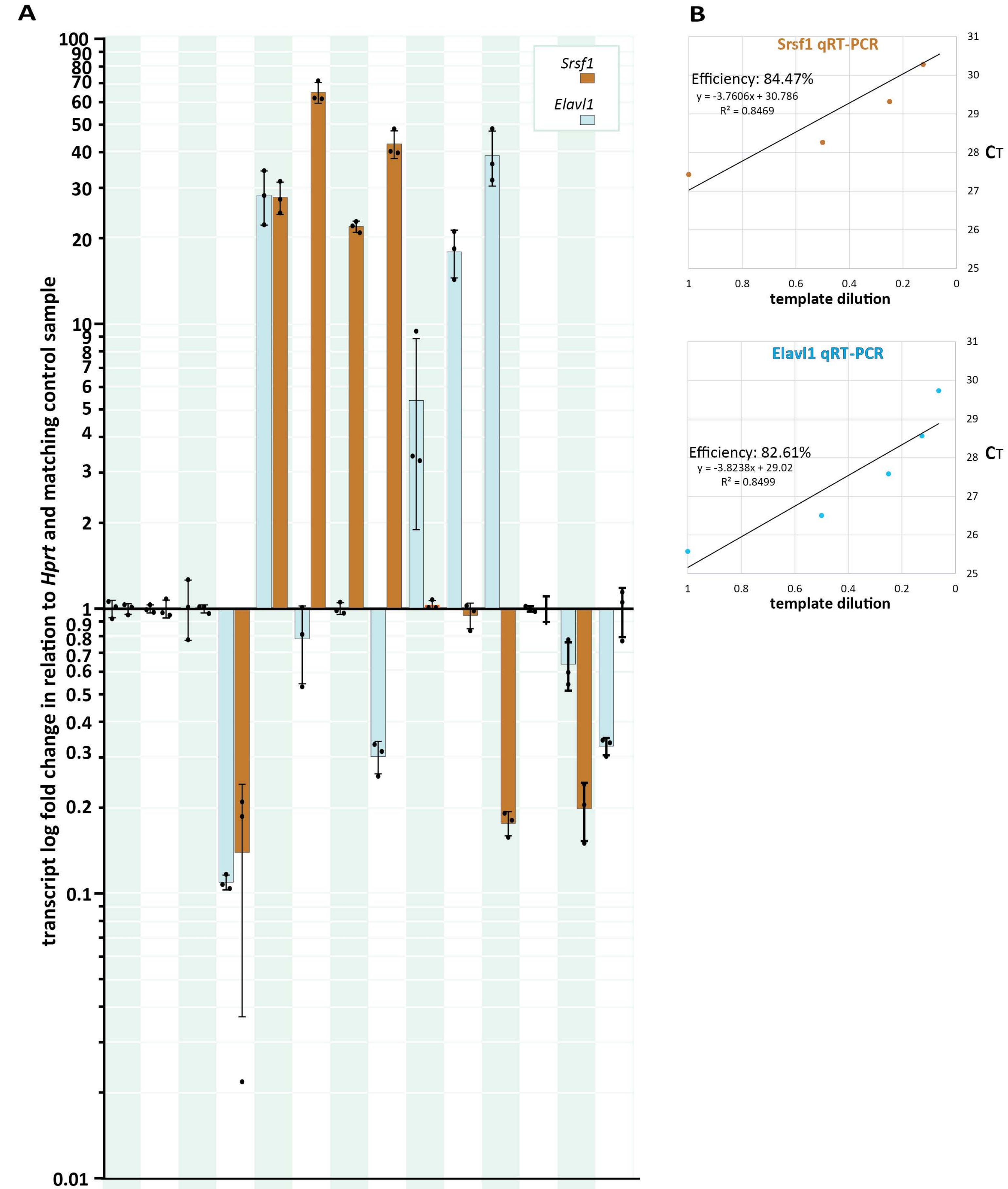

pCAGIG	−	+	+	−	−	−	−	−	−	−	−	−	−	−	pCAGIG
pCAG-Srsf1	−	−	−	−	+	+	+	+	−	−	−	−	−	−	pCAG-Srsf1
pCAG-Elavl1	−	−	−	−	+	−	−	−	+	+	+	−	−	−	pCAG-Elavl1
siCtrl	+	−	+	−	−	−	+	−	−	+	−	−	−	−	siCtrl
siSrsf1	−	−	−	+	−	−	−	−	−	−	+	−	−	−	siSrsf1
siElavl1	−	−	−	+	−	−	−	+	−	−	−	−	−	−	siElavl1
shScrambled	−	−	−	−	−	−	−	−	−	−	−	+	−	−	shScrambled
shSrsf1	−	−	−	−	−	−	−	−	−	−	−	−	+	−	shSrsf1
shElavl1	−	−	−	−	−	−	−	−	−	−	−	−	−	+	shElavl1

Supplementary figure 5 (pertaining to Figure 4)

A The result of modulating Srsf1 and Elavl1 transcript levels in N2a cells, quantified in relation to the transcript levels of *Hprt* and the matching control, that is, pCAGIG for overexpression samples (pCAG-Srsf, pCAG-Elavl1, or pCAG-Srsf+pCAG-Elavl1), siCtrl for simple knockdown samples (siSrsf1, siElavl1, or siSrsf1+siElavl1), shScrambled for shRNA samples (shSrsf1 or shElavl1), or pCAGIG+siCtrl for samples transfected with combinations of overexpression and knockdown constructs (pCAG-Srsf1+siCtrl or +siElavl1, pCAG-Elavl1+siCtrl or +siSrsf1).

B Comparison of efficiencies of the Srsf1 and Elavl1 RT-qPCR setups.

Supplementary figure 6
pertaining to Figure 5

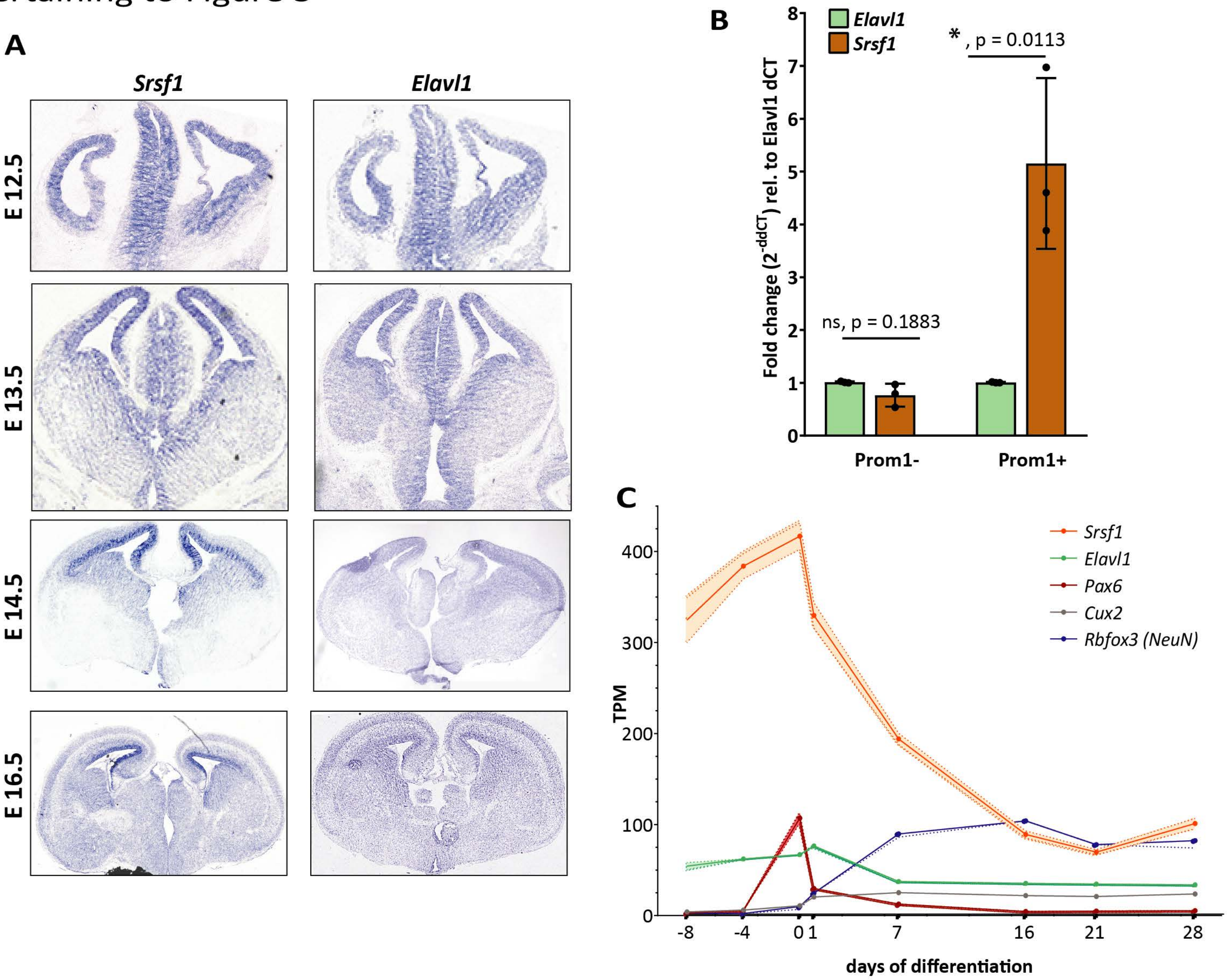

D

term	estimate	std.error	statistic	p.value
(Intercept)	0.095583	0.051758	1.84674	0.064785
**SRSF1**	0.149742	0.017679	8.469957	**2.45E-17**
**StageMouse E16**	0.344283	0.067174	5.125216	**2.97E-07**
**StageMouse E18**	-0.99386	0.087408	-11.3703	**5.88E-30**
CategoryPax6/Tbr2 Double Positive	0.110209	0.077567	1.420814	0.155371
**CategoryTbr2 Positive**	-0.21379	0.101367	-2.1091	**0.034936**
SRSF1:StageMouse E16	0.011707	0.022847	0.512403	0.608369
SRSF1:StageMouse E18	0.036123	0.080597	0.448197	0.654011
SRSF1:CategoryPax6/Tbr2 Double Positive	-0.03673	0.027667	-1.32752	0.184338
SRSF1:CategoryTbr2 Positive	0.044144	0.040336	1.094394	0.273782
**StageMouse E16:CategoryPax6/Tbr2 Double Positive**	-0.48313	0.104219	-4.63575	**3.56E-06**
StageMouse E18:CategoryPax6/Tbr2 Double Positive	-0.07054	0.182295	-0.38697	0.698777
**StageMouse E16:CategoryTbr2 Positive**	-0.52773	0.13383	-3.94326	**8.04E-05**
StageMouse E18:CategoryTbr2 Positive	0.321599	0.173403	1.854631	0.063649
SRSF1:StageMouse E16:CategoryPax6/Tbr2 Double Positive	0.045909	0.037247	1.232551	0.217743
SRSF1:StageMouse E18:CategoryPax6/Tbr2 Double Positive	0.335586	0.208905	1.606402	0.108186
SRSF1:StageMouse E16:CategoryTbr2 Positive	0.02597	0.05472	0.474595	0.635075
SRSF1:StageMouse E18:CategoryTbr2 Positive	0.004797	0.17866	0.026847	0.978581

E

Stage	p_value	n	p_value_adj
**Mouse E14**	**0.0003**	**1673**	**0.0009**
Mouse E16	0.2389	2042	0.2389
**Mouse E18**	**0.0009**	**1049**	**0.0014**

F

Stage	Category	p_value	n	p_value_adjusted
**Mouse E14**	**Pax6 Positive**	**0.005275**	761	**0.011867965**
Mouse E14	Pax6/Tbr2 Double Positi	0.425451	574	0.425450651
**Mouse E14**	**Tbr2 Positive**	**0.004518**	338	**0.011867965**
**Mouse E16**	**Pax6 Positive**	**0.000322**	963	**0.002899101**
Mouse E16	Pax6/Tbr2 Double Positi	0.172509	665	0.221797466
Mouse E16	Tbr2 Positive	0.254499	414	0.286311485
Mouse E18	Pax6 Positive	0.098383	690	0.147574834
**Mouse E18**	**Pax6/Tbr2 Double Positi**	**0.00515**	149	**0.011867965**
Mouse E18	Tbr2 Positive	**0.033861**	210	0.06095038

Supplementary figure 6 (pertaining to Figure 5)

A Panoramic captures of the brains resulting from RNA *in situ* hybridization for *Srsf1* and *Elavl1* depicted in Figure 4A.

B-C-The Srsf1 and Elavl1 transcripts are distributed in a cell type-specific manner in the developing neocortex.

B To address the expression of *Srsf1* and *Elavl1* in cortical neurons and NPCs, primary cortical cells from E 12.5 cortices were sorted for the Prom-1 antigen as described in Figure 1 C. RT-qPCR for *Srsf1* or *Elavl1* on RNA from the sorted cortical cells showed that Srsf1 transcripts are present in significantly higher amounts than Elavl1 transcripts in Prom-1-positive cells (apical radial glial NPCs) than in Prom-1-negative ones (neurons). Values depicted are fold changes in relation to *Elavl1* levels resulting from $2^{-\Delta\Delta C_T}$ analysis, setting the mean *Elavl1* levels in Prom-1-negative cells to 1. N=3. P values derived from unpaired, two-tailed Student's t test. Dots represent fold change values in individual replicates.

C Expression levels of *Srsf1* but not of *Elavl1* decrease sharply across neurodifferentiation of mouse embryonic stem cells (mESCs). TPM values were generated using Whippet(89) from the RNA sequencing data from (29). In the original data, the authors considered day -8 the day of culture start, whereas day 0 was the day from which cells were transferred to neuron differentiation medium. Also indicated are the levels of the progenitor marker *Pax6*, the intermediate (basal) progenitor marker *Cux2*, and the mature neuron marker *Rbfox3* (NeuN). N = 3-5 biological replicates for each time point. Dotted lines indicate SD values.

D Coefficients from fitting raw *Srsf1* and *Elavl1* expression values to a negative binomial model with "celltype" and "stage" as interaction terms.

E Summary of testing for stage-specific differences in *Srsf1* and *Elavl1* expression profiles for all progenitor types (Wilcoxon signed-rank paired test).

F Results of testing for differences in *Srsf1* and *Elavl1* expression profiles for each combination of stage and progenitor type (Wilcoxon signed-rank paired test).
